# Restoration of services in disrupted infrastructure systems: A network science approach

**DOI:** 10.1371/journal.pone.0192272

**Published:** 2018-02-14

**Authors:** Aybike Ulusan, Ozlem Ergun

**Affiliations:** Mechanical and Industrial Engineering Department, Northeastern University, Boston, Massachusetts, United States of America; Beijing Jiaotong University, CHINA

## Abstract

Due to the ubiquitous nature of disruptive extreme events, functionality of the critical infrastructure systems (CIS) is constantly at risk. In case of a disruption, in order to minimize the negative impact to the society, service networks operating on the CIS should be restored as quickly as possible. In this paper, we introduce a novel network science inspired measure to quantify the criticality of components within a disrupted service network and develop a restoration heuristic (Cent-Restore) that prioritizes restoration efforts based on this measure. As an illustrative case study, we consider a road network blocked by debris in the aftermath of a natural disaster. The debris obstructs the flow of relief aid and search-and-rescue teams between critical facilities and disaster sites, debilitating the emergency service network. In this context, the problem is defined as finding a schedule to clear the roads with the limited resources. First, we develop a mixed-integer programming model for the problem. Then we validate the efficiency and accuracy of the Cent-Restore heuristic on randomly generated instances by comparing it to the model. Furthermore, we use Cent-Restore to recommend real-time restoration plans for disrupted road networks of Boston and Manhattan and analyze the performance of the plans over time through resilience curves. We compare Cent-Restore to the current restoration guidelines proposed by FEMA and other strategies that prioritize the restoration efforts based on different measures. As a result we confirm the importance of including specific post-disruption attributes of the networks to create effective restoration strategies. Moreover, we explore the relationship between a service network’s resilience and its topological and operational characteristics under different disruption scenarios. The methods and insights provided in this work can be extended to other disrupted large-scale critical infrastructure systems in which the ultimate goal is to enable the functions of the overlaying service networks.

## Introduction

Critical infrastructure systems (CIS) underpin almost every aspect of the modern society by providing the essential functions through overlaying service networks. These service networks are commonly referred to as *lifelines* and considered vital such that their destruction would have a debilitating effect on economic productivity and daily living [1, 2]. Thus, after an extreme event disrupts them, it is important to restore the critical services provided by CIS as soon as possible, in order to minimize the negative impact to society. In this paper, we develop a generalizable measure and a framework to help stakeholders and managers of CIS to efficiently plan restoration operations so that the services provided within the system recover from unexpected major disruptions in a timely manner.

In this work, we focus on the disruptions created by natural hazards. For instance, after a natural hazard such as an earthquake, the transportation CIS is one of the many interdicted systems, mostly due to the accumulated debris that blocks the road network [3]. Blocked roads hamper the effectiveness of search-and-rescue operations, hinder the flow of relief aid to affected disaster sites, impede the evacuation of the disaster survivors, and constrain reachability to critical facilities. In short, the accessibility of the road network is degraded or destroyed [4]. In order to minimize the human losses, the initial response should reach the affected population within several days, rendering road restoration as one of the initial steps in disaster response and recovery [3, 5]. In the response phase following a disaster, road restoration consists of unblocking (clearing) roads by pushing the debris to the side.

In this context, we define an emergency service network overlaying on the physical road network that enables the flow of relief supplies and casualties between critical facilities (such as hospitals and distribution centers) and disaster sites. Due to the budget, resource, and time restrictions, complete recovery of the road network is often delayed [6]. As a result, in this study, the focus is to select a subset of disrupted roads and schedule them for recovery such that the functionality of the emergency service network would return to a stable level, where all the service demanding locations (disaster sites) are served, in the shortest possible time. As timeliness is the most significant concern, a recovery plan should aim to provide most of the service as early in the planning horizon as possible. We refer to this problem as the road network recovery problem (RNRP).

Network science is an emerging interdisciplinary field that includes practical and theoretical studies to enhance the understanding of complex networks [7]. It offers insightful information about a network’s behavior, such as its vulnerability (susceptibility to disruptions), robustness (ability to withstand disruptions), and fragility (inability to withstand disruptions). However, the integration of the understanding of networks through network science tools and the methods for optimizing network operations (mainly developed by operations researchers) is often overlooked and there exists a set of areas in which operations research community may be able to contribute to the network science field [8, 9], and vice versa. In this study, we use tools and insights developed by these two groups, namely the interaction of supply and demand over a service network and the topological properties of network components, to develop an effective restoration plan. In the rest of the paper, distribution of supply and demand points and their interaction with each other on a network will be referred to as the operational characteristics of a network.

We grouped the related literature into two streams: (i) papers that tackle problems broadly classified as network recovery problems; and (ii) papers that explore the effectiveness of network science measures in explaining the structure and post-disruption behavior of networks.

As the name suggests, the problem of network recovery focuses on operations that would repair the interdicted network components to bring the network back to its functional state. Due to the myriad amount of decisions involved, in general these problems are very complex [10]. Hence, in many papers, the problem is decomposed into two main decisions; network design and scheduling [11]. Network design decisions involve the selection of disrupted network components for recovery and scheduling decisions involve the ordering of these activities. In the literature, these decisions may be tackled either simultaneously, sequentially, or independently. In this paper, we consider these two decisions simultaneously.

There are several papers that only address the scheduling decision. Aksu et al. [12] present a path-based MIP model in order to maximize the dynamic (over-time) network accessibility by re-establishing links in a realistic road network. Given a pre-defined set of paths that should be accessible, their goal is to find an ordered sequence of the roads to recover. In addition, Chen et al. explore the problem of finding a schedule for the road recovery tasks [13]. They propose a two-level mathematical model, but due to the complexity of the model, they apply a metahueristic method (genetic algorithm) to solve the problem. Matisziw et al. develop a multi-objective MIP for the network recovery problem in which the goal is to reestablish the connectivity between origin-destination (o-d) pairs by repairing the disrupted arcs and nodes given a set of pre-defined o-d paths [6]. In this work, the amount of flow to send on an o-d path is set to be a parameter rather than a decision variable. In contrast, in this paper the network flows are incorporated as decisions. Other works that include the scheduling of recovery operations differentiate with respect to the assumptions and solution methodology employed [14–19].

The network recovery problem literature that considers the network design problem investigates the edges to be installed at each period over a planning horizon to optimize a certain objective and it is proven to be computationally hard to solve [20, 21]. A set of papers integrate network design and scheduling decisions to achieve an effective restoration planning [11, 22]. Cavdaroglu et al. develop a MIP model with the objective of minimizing costs and unmet demand [22]. Nurre et al. propose an integer programming formulation for the integrated network design and scheduling problem (INDS) [11]. The objective of this problem is to maximize the cumulative weighted flow arriving at the demand nodes. Similar to our work, in both of these papers, the objective is directly concerned with providing the service required in the demand nodes by reestablishing their connectivity with the supply locations. However, in the INDS problem [11], there is no explicit constraint limiting the recovery operations to the reachable arcs from the resources. Whereas, we restrict the recovery operations to reachable roads, which is aligned with real-time decision making.

The literature that fall into the second stream focus on network science methods to analyze the potential scope of the disruptive events on the networks. For instance, network vulnerability analysis measures the decrease in network functionality by removing vertices or edges by a variety of attack scenarios [23, 24], which is referred to as *attack vulnerability* in the literature. Attack scenarios include but are not limited to removing nodes or links based on random or network science measures such as degree, betweenness, and closeness centrality [23–28]. In order to assess the functionality of a network after an attack, the existence of paths between node pairs is checked through the emergence of the largest or the second largest component [23, 26, 29–31]. Hence, proper functioning of systems is assumed to entail connectivity of the network. However, we strongly emphasize that when there are other dynamics involved in the network, a highly fragmented network may also be fully functional [32]. For instance, in the problem of enabling the distribution of relief aid from supply locations to demand locations, connectivity of demand to any supply with enough capacity is sufficient for full functionality, which may result in several small components. As opposed to most of the network science literature, we assess the functionality of a network based on demand satisfaction over the planning horizon and demonstrate it through a graphical curve which we refer as the *resilience curve*. We refer the reader to Bruneau et al. for a detailed discussion on resilience and how it can be evaluated [33].

The literature is limited when it comes to utilizing network science methods in restoration. Bhatia et al. propose recovery strategies for large-scale CIS through network centrality measures [34]. However, they do not include the service component into the problem. When the goal is set as re-establishing the functionality of the service network rather than the complete recovery of CIS, generic network science measures fall short in explaining the dynamics of the networks. In our work we discuss these shortcomings and introduce a new network science measure.

In summary, the contribution of this paper is threefold: (i) proposing a new network science measure for assessing the criticality of disrupted service network components; (ii) developing a network science based restoration heuristic for service networks; (iii) exploring the relationship between service networks’ different characteristics and their resilience under various disruption scenarios to derive insights on resilience.

## Materials and methods

### Road network recovery problem

We model RNRP as a multi-period MIP model on an undirected network *G*(*N*, *E*). There are two subsets in the node set *N* is divided into three subsets: *N*_*S*_ indicates the set of supply nodes, critical facilities such as hospitals, distribution centers, shelters, *N*_*D*_ indicates the set of demand nodes, disaster sites composed of the people in need for relief commodities and services, and the remaining nodes that are neither a supply nor a demand node. The edge set *E* refers to the roads in the network, composed of debris-blocked roads, $\bar{E}$, and unblocked roads, $E\setminus \bar{E}$. The resources required for road recovery are clearance resources and their amount is limited at each time period, denoted by *R*_*t*_. As a result, the planning horizon, *T*, is divided into discrete time periods, *t* ∈ *T*, and the model parameters and variables are defined for each time period. The clearance resources are assumed to be located at the supply nodes in the beginning of the planning horizon. Note that, the resource allocation decision is outside the scope of this work, hence at each time period the resources are defined as an aggregate value reachable from all supply locations. Similar works focusing on the scheduling of the restoration activities also define an aggregate value for resources and omit the resource allocation decision [11, 12, 28]. Furthermore, the clearance resources can-not traverse debris blocked roads, hence a blocked road has to be reachable through an unblocked path from a clearance resource in order to be cleared. Time to traverse an unblocked road and time to reestablish the service between supply and demand nodes is very short compared to the time to clear the debris and recover the roads. Thus traversal times are assumed to be negligible and not included in the model, and as soon as the connectivity is established with a demand point, supply is allocated immediately within the same time period. In addition, we assume that the amount of debris accumulated on the roads is known, and for simplicity we assume that a unit of debris requires a single clearance resource to be cleared. We refer the reader to S1 Table for the detailed explanation of the notation used in the formulation of the problem.

Each supply node *i* ∈ *N*_*S*_ has an initial supply capacity of $rs_{i}^{0}$, and each demand node *j* ∈ *N*_*D*_ has an initial demand of $ud_{j}^{0}$. At the end of period *t*, $rs_{i}^{t}$ and $ud_{j}^{t}$ indicate remaining supply capacity at node *i* ∈ *N* and remaining unsatisfied demand at node *j* ∈ *N*, respectively. Without loss of generality, we assume that $rs_{i}^{t}$ is equal to 0 for *i* ∈ *N* \ *N*_*S*_ and $ud_{j}^{t}$ is equal to 0 for *j* ∈ *N* \ *N*_*D*_. Once a demand node gets connected to a supply node through an unblocked path, flow between supply and demand nodes begin. If there is enough supply, the amount of flow is equal to the amount of demand, otherwise it is equal to available supply. Without loss of generality, we assume the flow is in the direction from supply nodes to the demand nodes. Consequently, the amount of unsatisfied demand decreases by the amount of the incoming flow, and the available supply of the supply node allocating the flow is decreased by the outgoing flow. Furthermore, we assume that an external factor can not add to or diminish the unsatisfied demand or the available supply in the middle of the planning horizon.

The mixed-integer programming formulation for the RNRP is given as follows:

$$\begin{array}{cc} \max\; & \sum_{t=1}^{T}b^{t}\sum_{i\in N_{D}}d_{i}^{t} \end{array}$$ (RNRP-MIP)

subject to

$$\begin{array}{cc} {ud}_{i}^{t-1}-{ud}_{i}^{t}=d_{i}^{t}, & \forall i\in N,\forall t\in T, \end{array}$$ (1)

$$\begin{array}{cc} {rs}_{i}^{t-1}-{rs}_{i}^{t}=s_{i}^{t}, & \forall i\in N,\forall t\in T, \end{array}$$ (2)

$$\begin{array}{cc} \sum_{(j,i)\in E}f_{j,i}^{t}-\sum_{(i,j)\in E}f_{i,j}^{t}=d_{i}^{t}-s_{i}^{t}, & \forall i\in N,\forall t\in T, \end{array}$$ (3)

$$\begin{array}{cc} f_{i,j}^{t}\leq \gamma_{i,j}^{t}M, & \forall (i,j)\in E,\forall t\in T, \end{array}$$ (4)

$$\begin{array}{cc} A_{i,j}^{t-1}+y_{i,j}^{t}=A_{i,j}^{t}, & \forall \left(i,j\right)\in \bar{E},\forall t\in T, \end{array}$$ (5)

$$\begin{array}{cc} W_{i,j}-A_{i,j}^{t}\leq \left(1-\gamma_{i,j}^{t}\right)W_{i,j}, & \forall \left(i,j\right)\in \bar{E},\forall t\in T, \end{array}$$ (6)

$$\begin{array}{cc} W_{i,j}-A_{i,j}^{t}\geq \left(1-\gamma_{i,j}^{t}\right), & \forall \left(i,j\right)\in \bar{E},\forall t\in T, \end{array}$$ (7)

$$\begin{array}{cc} \sum_{\left(i,j\right)\in \bar{E}}y_{i,j}^{t}\leq R_{t}, & \forall t\in T, \end{array}$$ (8)

$$\begin{array}{cc} y_{i,j}^{t},A_{i,j}^{t}\geq 0, & \forall \left(i,j\right)\in \bar{E},t\in T, \end{array}$$ (9)

$$\begin{array}{cc} \gamma_{i,j}^{t}\in \left\{0,1\right\}, & \forall \left(i,j\right)\in \bar{E},t\in T, \end{array}$$ (10)

$$\begin{array}{cc} {ud}_{i}^{t},{rs}_{i}^{t},d_{i}^{t},s_{i}^{t}\geq 0, & \forall i\in N,\forall t\in T \end{array}$$ (11)

$$\begin{array}{cc} f_{i,j}^{t}\geq 0, & \forall (i,j)\in E,\forall t\in T. \end{array}$$ (12)

The objective function of RNRP-MIP is to maximize the total benefit accrued by satisfying demand over time. At each period *t*, a unit of satisfied demand will contribute a weight of *b*^*t*^ to the objective function. These weights are an output of the benefit function which decreases non-linearly throughout the planning horizon and is interpreted as a measure of the disaster survivors’ well-being. The decreasing nature of the function creates an incentive for demand satisfaction in the earlier periods, which is necessary since the first several days after a disaster are crucial and referred as the *golden period* [5, 10, 12]. In addition, such a function is expected to be monotonic and convex over time [35]. For the RNRP, we define this function as exponentially decreasing over time (see S1 Fig),

$$\begin{array}{c} b^{t}=Ce^{-\lambda t}. \end{array}$$

As the value of the parameter λ increases, the slope of the decline in the benefit function increases, leading to more aggressive decreases in the early periods. For RNRP-MIP, we set the value of λ as 0.2 and *C* to 120. In our initial experiments, these numerical values provide plausible results, however, depending on the decision maker’s preferences and interpretation of the urgency of restoration efforts, different values may be picked.

Constraints (1) and (2) specify the dynamics of demand satisfaction and supply allocation, respectively, throughout the planning horizon. Constraint (3) establishes the flow between supply and demand nodes through a set of nodes and edges. If an edge is not completely recovered, then there shouldn’t be any flow on it, which is ensured with constraint (4). The progress of the clearance activities is demonstrated with constraint (5). At the end of each period, the amount of total clearance activity completed for an edge {*i*, *j*}, $A_{i,j}^{t}$, is calculated by simply adding clearance activity done at the current period, $y_{i,j}^{t}$ to the total clearance completed until the end of previous period, $A_{i,j}^{t-1}$. Constraints (6) and (7) specify the period when an edge will be labeled as fully cleared. When the amount of clearance activity completed until the end of time $t,A_{i,j}^{t}$ is equal to the required amount of clearance that should be performed for full recovery, *W*_*i*,*j*_, variable $\gamma_{i,j}^{t^{\prime }}:t^{\prime }\geq t$ is set to 1, rendering edge {*i*, *j*} available for sending flow starting from period *t*. Without loss of generality, we assume $\gamma_{i,j}^{t}$ is equal to 1 for *t* ≥ 0 if edge $\left\{i,j\right\}\in E\setminus \bar{E}$. Finally, constraint (8) restricts the amount of clearance resources that are available for recovery at a particular time period. Constraints (9)-(12) are the integrality and non-negativity constraints for the decision variables.

As mentioned before, in order for the clearance teams to reach a blocked edge, there should be an unblocked path from the clearance teams’ location to that particular blocked edge. We next prove that such a solution can always be found in the optimal solution set of RNRP-MIP without explicit constraints.

**Theorem 1.** For any optimal solution to RNRP-MIP, consisting of a set of restoration activities to be performed in each discrete time period *t* during the planning horizon T, there exists an equivalent optimal solution where only the roads that are reachable from a clearance resource are cleared.

*Proof.* Proof of Theorem 1 is included in S1 Appendix.

### Heuristic-Based solution approach

State-of-the-art optimization solvers can solve small-sized instances of RNRP-MIP to optimality, but in order to solve moderate to large-sized instances we develop a heuristic to find a set of restoration activities performed (which consists of the cleared roads and the amount of clearance) at each discrete time period. We propose a new centrality measure that assesses the benefit of recovering the disrupted network components in a service-oriented environment to use it in a restoration heuristic. While developing the new centrality measure, we build upon the widely used network science measure, betweenness centrality measure.

#### Generic betweenness centrality

Betweenness centrality was first proposed by Freeman [36] to evaluate the importance of the network nodes based on the frequency of times they lie on the shortest paths between other nodes [37]. For example, consider a transportation CIS in which vehicles are moving from one intersection point (node) to another and always preferring the shortest distanced (geodesic) paths. Hence, the number of times that a vehicle passes by a node is proportional to the number of geodesic paths that this node lies on.

Let *g*_*ij*_ denote the number of geodesic paths between nodes *i* and *j*, and let $g_{ij}^{k}$ indicate the number of geodesic paths between *i* and *j* that go through node *k*. Then the betweenness centrality of node *k*, *C*_*k*_, is the sum of the ratio of the geodesic paths that pass through *k* to the number of all geodesic paths between *i* and *j* over all node pairs {*i*, *j*}:

$$\begin{array}{c} C_{k}=\sum_{i\in N}\sum_{j\in N}\frac{g_{ij}^{k}}{g_{ij}}. \end{array}$$

In this formula we adopt the convention that the fraction $g_{ij}^{k}/g_{ij}$ equals to zero if both $g_{ij}^{k}$ and *g*_*ij*_ are equal to zero.

Nodes with high betweennness centrality may have considerable influence within a network since they lie on the largest number of paths used. Thus, any disruption to them will hamper the flow between most of the nodes [37], leading to fragmentation of the network and a decrease in its functionality. This statement has also been proven in the attack vulnerability literature. For instance, Guimera et al. showed that for a world-wide airport network, nodes with high betweenness centrality are the most important nodes in keeping the network connected [38].

Symmetrically, we claim that the restoration of edges leading to a node having a high betweenness centrality will result in the greatest improvement in network’s functionality. However, when the network functionality is not merely evaluated on the network connectivity, but on the flow of the services, the generic betweenness centrality measure falls short, leading to the need for a new measure.

#### A new betweenness centrality measure - SNEBC

We develop a new measure to assess the importance of the disrupted edges in a service network and call this measure the service network edge betweenness centrality (SNEBC). We construct SNEBC through five main modifications to the generic node betweenness centrality measure and below we describe these modifications in the context of RNRP. Nevertheless, the discussion can be adopted to any disrupted service network scenario by considering the debris amounts as the disruption intensity, supply and demand nodes not only as hospitals and disaster survivors but any location providing and requiring service, and clearance resources as the restoration capacity in a given time period.

In RNRP, the time to traverse the roads is negligible compared to the time to clear the debris off the roads. As a result, the shortest path is calculated based on a road’s debris amounts rather than its metric distance. Hence, we set the first modification as preferring the paths that have minimal debris rather than shortest in metric distance when computing the centrality of the nodes.

The demand of the disaster survivors is served through first re-establishing their connectivity to hospitals (supply nodes). Therefore, instead of calculating how often a node lies on the shortest paths between all node pairs, we calculate how often a node lies on the shortest paths between supply and demand nodes. In other words, the second modification on the measure entails taking out the possibility of nodes being evaluated based on non-essential paths they lie on.

As an extension to the first two modifications, the shorter paths should be more important, since they reach demand nodes faster. Hence, we express the third modification as an emphasis on the paths that are shorter by simply scaling the centrality measure with the weight of the shortest paths. Putting the first three modifications together, the updated betweenness centrality measure is expressed as,

$$\begin{array}{c} {\hat{C}}_{k}=\sum_{i\in N_{S}}\sum_{j\in N_{D}}\frac{{\bar{g}}_{ij}^{k}}{{\bar{g}}_{ij}D_{ij}}, \end{array}$$

where ${\bar{g}}_{ij}$ denotes the number of shortest paths (calculated based on the debris amounts) from *i* ∈ *N*_*S*_ to *j* ∈ *N*_*D*_, and ${\bar{g}}_{ij}^{k}$ is the number of times node *k* belongs to these shortest paths. Weight (total debris amount) of the shortest path between nodes *i* and *j* is equal to *D*_*ij*_.

Furthermore, the supply and demand amounts of the nodes vary, so the paths connecting different supply and demand nodes should be differentiated. For example, the paths originating from larger supply nodes and paths that lead to larger demand nodes should be more important in the restoration activities, so that these nodes can be reached earlier in the planning horizon. A particular complexity associated with such a prioritization scheme occurs when the amount of available clearance resources in the current period is not enough to clear all the debris on the path to reach the large demand nodes. In this case, a better action for the current period may be to prioritize a road that can be cleared in the current period but leads to a smaller demand node. As a result, an immediate benefit is accrued for satisfying a demand in the current period. However, this action might adversely affect the later periods, so it is not straightforward to establish the trade-off between these two actions. We define a path weight function $\phi (ud_{j}^{t},rs_{i}^{t})$ in Eq (13) that captures this trade-off to some extent:

$$\begin{array}{c} \phi \left(ud_{j}^{t},rs_{i}^{t}\right)=\left\{ \begin{array}{cc} rs_{i}^{t}ud_{j}^{t}\left[1-(\frac{1}{t}-\frac{1}{t+1})\right], & \text{if}\;D_{ij}>{\bar{R}}_{t}\\ rs_{i}^{t}ud_{j}^{t}, & \text{otherwise}. \end{array}\right. \end{array}$$ (13)

The path weight function is defined to be increasing with increasing $ud_{j}^{t}$ and $rs_{i}^{t}$ values. When the remaining resource amount in the current period *t*, ${\bar{R}}_{t}$, is not enough to recover a path leading to demand node *j*, the weight of the path is decreased by a time period dependent amount. This decrease is larger in the early periods, giving more importance to smaller but reachable demand in the current period. As a result, the paths leading to large demand nodes are still prioritized, but if the resources required to clear a road are more than the available resources, a greedier approach leading to larger demand nodes is adopted.

Taking all the modifications into consideration, the new service network betweenness centrality measure (SNBC), for a node *k* is given by,

$$\begin{array}{c} {\bar{C}}_{k}=\sum_{i\in N_{S}}\sum_{j\in N_{D}}\frac{{\bar{g}}_{ij}^{k}}{{\bar{g}}_{ij}}\frac{\phi (ud_{j}^{t},rs_{i}^{t})}{D_{ij}}. \end{array}$$ (SNBC)

Since RNRP seeks to find the sequence of roads (edges) to be restored throughout the planning horizon, SNBC, which is a measure for the nodes, is converted into a service network edge betweenness centrality (SNEBC) by adding the SNBC of an edge’s end nodes. For an edge {*k*, *l*}, it can be expressed as,

$$\begin{array}{c} {\bar{C}}_{kl}={\bar{C}}_{k}+{\bar{C}}_{l}. \end{array}$$ (SNEBC)

#### SNEBC-based restoration heuristic for RNRP (cent-restore)

The heuristic we develop to determine scheduling of restoration activities on a disrupted service network has the following steps.

**Input:** Disrupted service network: *G*(*N*, *E*)

   Debris amount for each disrupted road $\left\{k,l\right\}\in \bar{E}$: *W*_*k*,*l*_

   Supply and demand amounts on the nodes

   Available clearance resources at each time period *t*: *R*_*t*_

**Initialization:** *t* ≔ 1

$S_{t}:=\emptyset ,\;\forall t\in T$   ($S_{t}$: set of partially or completely cleared roads at time *t*)

$w_{k,l}^{t}:=0,\;\forall t\in T,\;\forall \left\{k,l\right\}\in \bar{E}$  ($w_{k,l}^{t}$: Amount of clearance performed

                     on road $\left\{k,l\right\}\in \bar{E}$ at time *t*)

${\bar{W}}_{k,l}:=W_{k,l},\forall \left\{k,l\right\}\in \bar{E}$    (${\bar{W}}_{k,l}$: Remaining debris on road $\left\{k,l\right\}\in \bar{E}$)

*z*_*H*_ ≔ 0               (*z*_*H*_: Objective value)

1. Calculate SNEBC, ${\bar{C}}_{kl}$, for all the disrupted roads $\left\{k,l\right\}\in \bar{E}$ that are reachable from any clearance resource at period *t*
2. Sort the roads in descending order of SNEBC (break ties in favor of the roads with less debris) and select the first road, {*k*, *l*}, from the list
3. Add {*k*, *l*} to $S_{t}$
  3.1 **If** ${\bar{W}}_{k,l}\leq R_{t}$,
    - Road {*k*, *l*} is fully cleared ($\bar{E}:=\bar{E}\setminus \left\{k,l\right\}$)
    - $w_{k,l}^{t}:={\bar{W}}_{k,l}$
    - $R_{t}:=R_{t}-w_{k,l}^{t}$
    - Identify the demand nodes that connect to a supply node after road {*k*, *l*} is cleared
    - Immediately satisfy the newly connected demand from the accessible supply nodes and update the remaining demand and supply amounts on the nodes, add the benefit from satisfying the demands at time *t* to the objective *z*_*H*_
      ⋅ Note that if the connected supply is not sufficient to satisfy all the demand on the recently connected nodes, satisfy as much demand as possible and leave the unfulfilled demand on any demand node. Since all the demand is connected with unblocked edges, it does not make any difference which demand node is left unsatisfied.
    **If** all the demand in the network is satisfied,
      ⋅ Terminate the process
      ⋅ **Return** *z*_*H*_, $S_{t},w_{k,l}^{t}\forall t\in T,\;\forall \left\{k,l\right\}\in \bar{E}$
    **Else**
      ⋅ **If** *R*_*t*_ = 0, then *t* ≔ *t* + 1
      ⋅ Go back to step (1) and repeat the process with the updated demand, supply, and unblocked and blocked roads’ information
  3.2 **Else**
    - Road {*k*, *l*} is partially cleared with the available resources at time *t*, *R*_*t*_
    - $w_{k,l}^{t}:=R_{t}$
    - ${\bar{W}}_{k,l}:={\bar{W}}_{k,l}-w_{k,l}^{t}$
    - *R*_*t*_ ≔ 0
    - *t* ≔ *t* + 1
    - Go back to step (1) and repeat the process with the updated remaining debris information, ${\bar{W}}_{k,l}$

When a road {*k*, *l*} is selected for clearance at time *t*, it is automatically added to the set $S_{t}$, and the amount of clearance that can be performed on that road (depending on the available resources) is stored in $w_{k,l}^{t}$. Consequently, the amount of available resources at *t* is diminished by the amount of clearance performed. If the available resources are not completely depleted, a new road to clear at time *t* is sought, otherwise, $S_{t}$ results as the set of roads considered for clearance at time *t*, and the process moves on to the next time period *t* + 1 to fill up $S_{t+1}$.

Note that, the first step is performed many times until termination, and computing the SNEBC values for every road at each step is computationally inefficient since it involves the computation of all the shortest paths between all supply-demand node pairs and detecting the occurrence of particular nodes on paths. We employ several strategies to decrease the computational burden. First, we use the widely adopted vertex collapse technique to reduce the network size drastically [39, 40]. By this technique, the nodes that are connected with unblocked roads are collapsed into a super-node and the blocked roads are kept as edges connecting the super-nodes. If parallel edges exist between super-nodes, only the edge with the least debris is kept so that there is a single edge connecting each node pair. Next, the calculation of shortest paths is performed using a Fibonacci heap structure in the implementation of Dijkstra algorithm. Finally, we employ the methodology proposed by Newman [37], which traces back the shortest path tree to calculate node betweenness centrality based on the shortest path information.

### Computational study setup

The goals of the computational study are threefold: (i) analyzing the accuracy and efficiency of Cent-Restore with respect to optimal solutions obtained by solving RNRP-MIP; (ii) examining the impact of networks’ operational and topological properties on their resilience; and (iii) comparing the performance of the developed heuristic with other prioritization schemes for restoration efforts.

**Fig 1:**
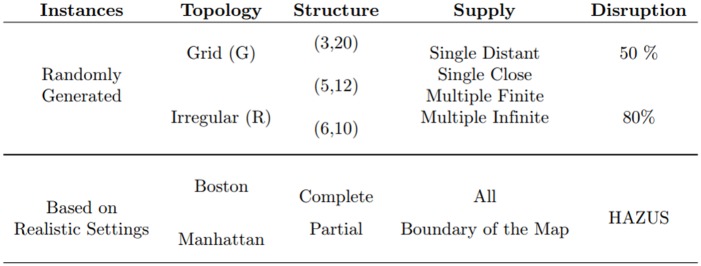
Summary of the instances. Instances are divided into two categories based on the way they are generated: random or based on realistic settings. If they are randomly generated, they are further divided into two based on their topology: grid or irregular (resembling a random planar network), then divided into three based on their structure (height x width), and into four, varying based on the supply capacity and locations. The random disruption results in either 50% or 80% blockage of the roads. For realistic settings, we use Boston and Manhattan and either define the instances on a partition of the maps (Partial Instances) or on the entire map (Complete Instances). For the complete instances, two supply settings are defined: either with all the hospitals, or with just the hospitals on the boundary of the maps being available. For the partial instances, all the hospitals in that partition are selected to be available. The earthquake scenarios are simulated with HAZUS [41].

We group the instance set under two main categories: randomly generated instances and instances based on realistic settings. Under each category, we further classify the networks based on their topology. Moreover, for some settings we perform additional testing by varying the quantity and location of supply nodes, disruption intensity, and other structural attributes of the networks. Fig 1 demonstrates a high-level grouping of the instances used in the computational experiments. In the subsequent sections we give detailed explanation of the instance features.

We create instance types by combining features from each column of Fig 1. An example of an instance type for a randomly generated instance may be an irregular network (R) with topology (5, 12) having multiple supplies with finite capacity and a disruption on 50% of its edges.

#### Randomly generated instances

Let grid network *G*(*m*, *n*) represents a graph with *N*(= *m* ∗ *n*) equidistant nodes that are positioned in a rectangular area of height *m* and width *n*. We fix the number of nodes to *N* = 60, and define three rectangular areas (height x width) to position the nodes: 3x20, 5x12, 6x10, and will refer to them as the structure of the instance types. As the width and height of the rectangle gets closer, average topological distance between the nodes decreases. As a result, the diameter that refers to the maximum shortest path between any pair of nodes decreases. Hence, among the 3 different structured networks, 3x20 has the largest diameter.

**Fig 2:**
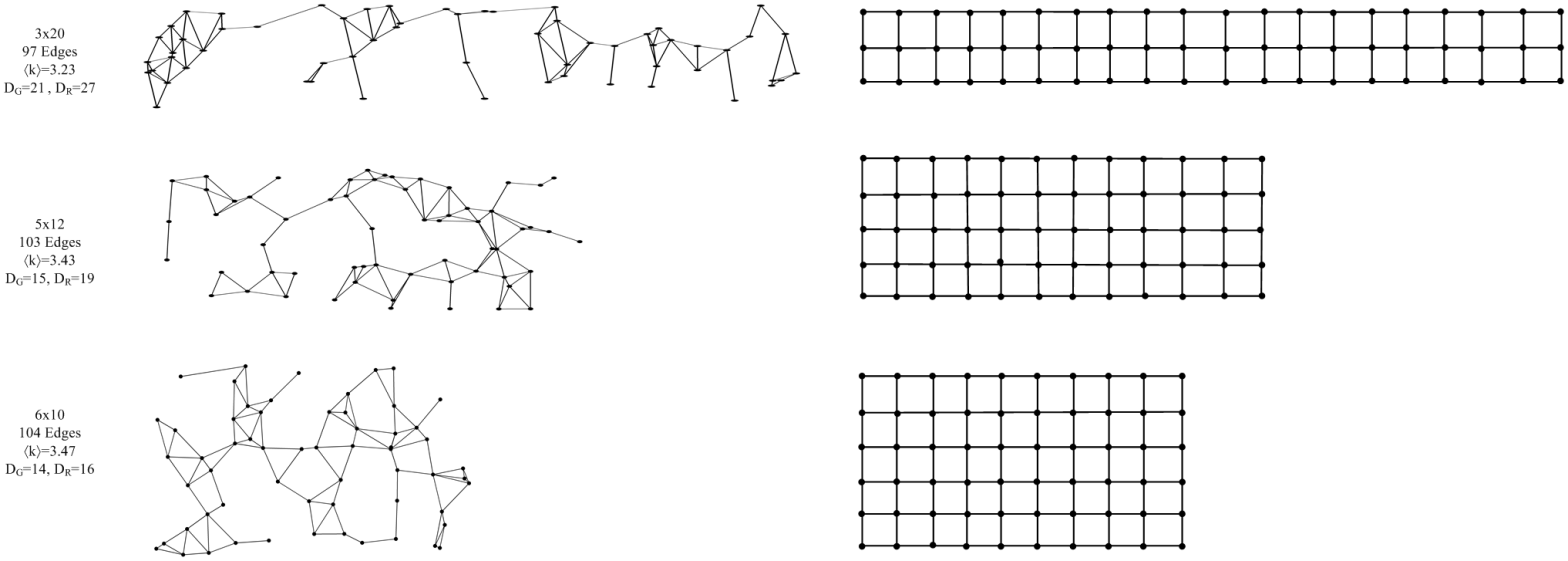
Illustration of the generated irregular (left panel) and grid (right panel) networks with different diameters. The diameter of the networks decreases from top to bottom. *D*_*G*_ and *D*_*R*_ indicates the diameter of the grid and irregular networks, respectively. 〈*k*〉 indicates the average degree.

On the other hand, *R*(*m*, *n*) represents an irregular graph with *N*(= *m* ∗ *n*) nodes randomly positioned in a rectangular area of height *m* and width *n*. We define the same three structures as grid networks, 3x20, 5x12, 6x10 to position 60 nodes. In addition, we set the number of edges equal to the number of edges of grid networks that have the same structure, so that we can compare the two instance types properly. As a result, the average degree for the two instance types with the same structure is the same. Fig 2 demonstrates the structure and some of the topological properties of the grid and irregular networks.

The detailed process we used for building the grid and irregular networks (referred to as Erdös-Rényi Planar Graph) can be found in the literature [42, 43].

As Fig 1 suggests, we generate four supply scenarios: (i) Single Distant, single supply point with a large or infinite capacity, positioned on the node with the largest average metric distance from all the other nodes, (ii) Single Close, single supply point with a large or infinite capacity, positioned on the node with the smallest average metric distance from all the other nodes, (iii) Multiple Finite, multiple supply points located randomly, each having the same finite capacity (total supply capacity is set to be just enough to satisfy the total demand), (iv) Multiple Infinite, multiple supply points located randomly, all having a large or infinite capacity.

We refer the reader to S2 Appendix for a detailed explanation on how the parameters such as debris amounts, clearance resources, and demand nodes are randomly set for different instance types.

#### Instances based on realistic settings

Over time, urban cities evolved in two distinct ways; self-organized and planned [44]. For the analysis of instances based on realistic settings, we select one city representing each category. Manhattan, widely known by its grid-patterned road network, is selected for representing a planned city [30, 45]. On the other hand, Boston is selected for representing a self-organized city (irregular network) due to the lack of city planning in most of its areas [46].

**Fig 3:**
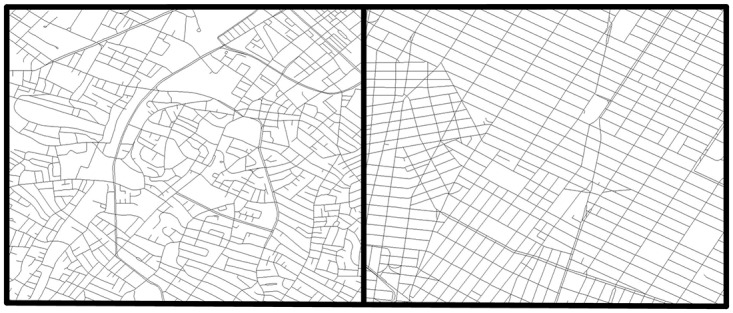
Examples of road network patterns. Boston with an irregular road network (left panel) and Manhattan with a grid-like road network (right panel).

We extract the road networks for Boston and Manhattan from the Massachusetts GIS [47] and New York State GIS Clearinghouse [48], respectively. Next, we clean the extracted networks based on their road features. For instance, any isolated road which renders the network disconnected, thoroughfares such as walkways, stairways, driveways, parking areas, docks, bicycle roads etc. are removed from the data. A localized view of the road patterns for two cities is shown in Fig 3.

**Fig 4:**
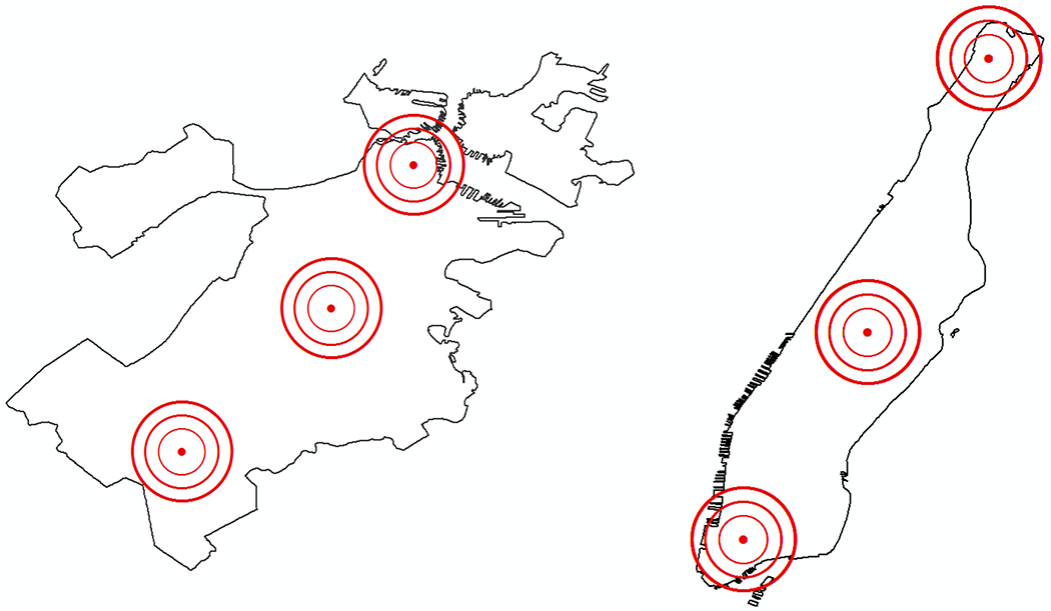
Epicenters of 3 earthquake scenarios on maps of Boston (left panel) and Manhattan (right panel). All three earthquake scenarios have a magnitude of 7.0 and a depth of 10 km.

First, we use disaster simulation software HAZUS of Federal Emergency Management Agency (FEMA) to estimate debris amounts on the roads by generating different disaster scenarios [41]. Fig 4 demonstrates three different earthquake scenarios with varying epicenters but having the same depth (10 km) and magnitude (7.0). The earthquake model HAZUS provides has a sophisticated debris estimation tool, which has been proven to provide accurate estimates [49]. As an outcome of the disaster simulation, it provides the debris estimates on the census tracts. In order to generate the debris amounts on the road segments, we first classify roads based on their census tracts and distribute the aggregate debris on the corresponding tracts proportional to the roads’ physical lengths. When the estimated amount of debris on a road is small enough, i.e. if it takes less than 10 minutes to be cleared by the available clearance resources, that debris is omitted, and the road is assumed to be unblocked.

As Feng et al. suggest, we assume the clearing rate of a clearance team (resource) to be 250*m*^3^/hr [5], and we assume that the clearance teams work 10 hr/day on average. HAZUS provides the debris estimates in terms of mass (tons), thus we convert the volume-based clearance rate (*m*^3^/hr) to a mass-based rate (tons/hr) using guidelines provided by FEMA [50]. We set different values for the number of clearance teams for each disaster scenario so that the ratio of the total clearance ability of the resources to the total debris amount generated by each disaster scenario would be the same, and as a result the comparison between different scenarios will not be biased. Having at most 750 clearance teams is a reasonable assumption [49]; in this work we relaxed this assumption slightly to having at most ≈800 clearance teams.

We generate demand amounts on nodes based on the population and debris intensity on census tracts. We associate a unit of demand with a single person. First, we generate an aggregate demand value at each census tract by scaling the census tract populations (retrieved from HAZUS’ database) with respect to the debris amount on that tract. For instance, in the areas where debris amount is low, the number of people with demand will be low. This number grows exponentially as the debris amount increases. Then, for each tract we randomly select a set of nodes and associate a demand amount with them in such a way that the demand value on any particular node would be at least five people.

**Fig 5:**
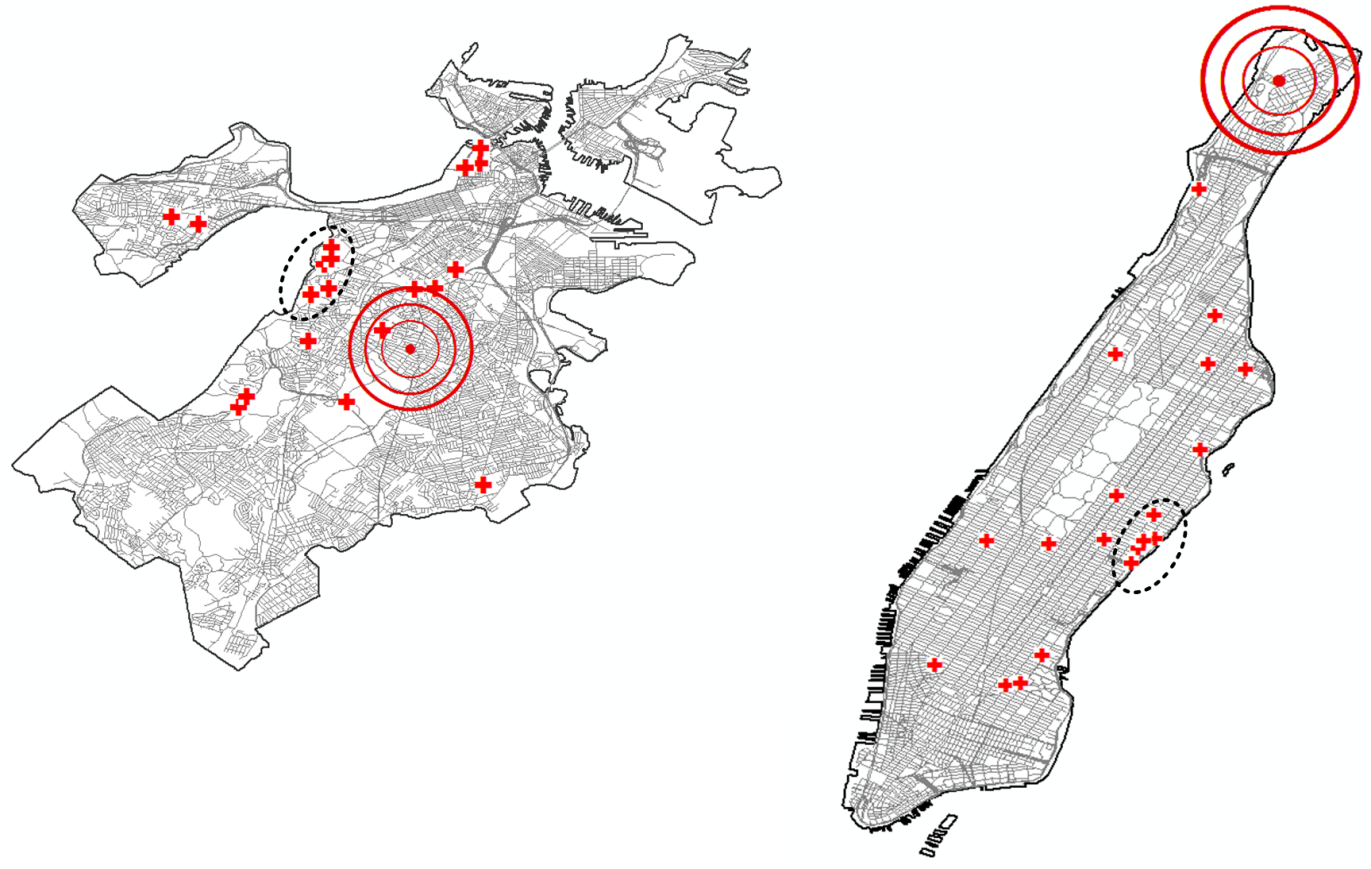
Road networks of Boston and Manhattan with all the hospitals and a sample earthquake scenario. We simulate a sample earthquake scenario with the epicenter as indicated, having a depth of 10 km and a magnitude of 7.0. We determine a subset of the hospitals that are close to the map boundary as indicated by the dashed ellipsoid on the maps. Maps are composed in ESRI ArcGIS 10.2.2.

Hospital locations and their capacities are also retrieved from the HAZUS database. We assume that the total supply capacity is at least equal to the amount of total demand, thus we scale the given capacities of hospitals so that the total supply is just enough to cover all the demand but is not excessive. In order to compare instances properly, we fix the ratio of total demand to supply. As mentioned in Fig 1, in addition to conducting experiments with all the hospitals, we generate a new scenario keeping the hospitals close to the boundaries of the maps. The hospitals in this supply scenario are marked with a dashed ellipse of Fig 5.

**Fig 6:**
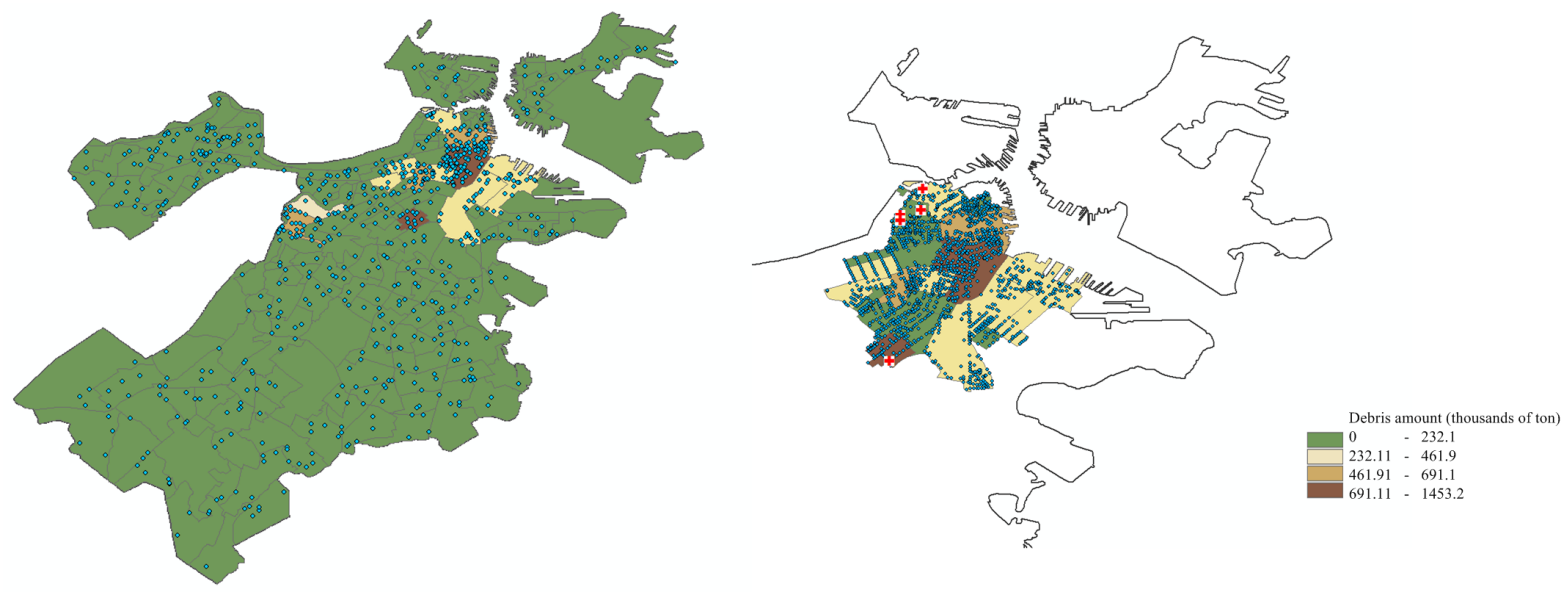
Boston after disruption. Debris amounts on the census tracts and resulting demand nodes from the sample earthquake in Fig 5 demonstrated on the middle and right panel (partial Boston instance), respectively. Maps are composed in ESRI ArcGIS 10.2.2.

**Fig 7:**
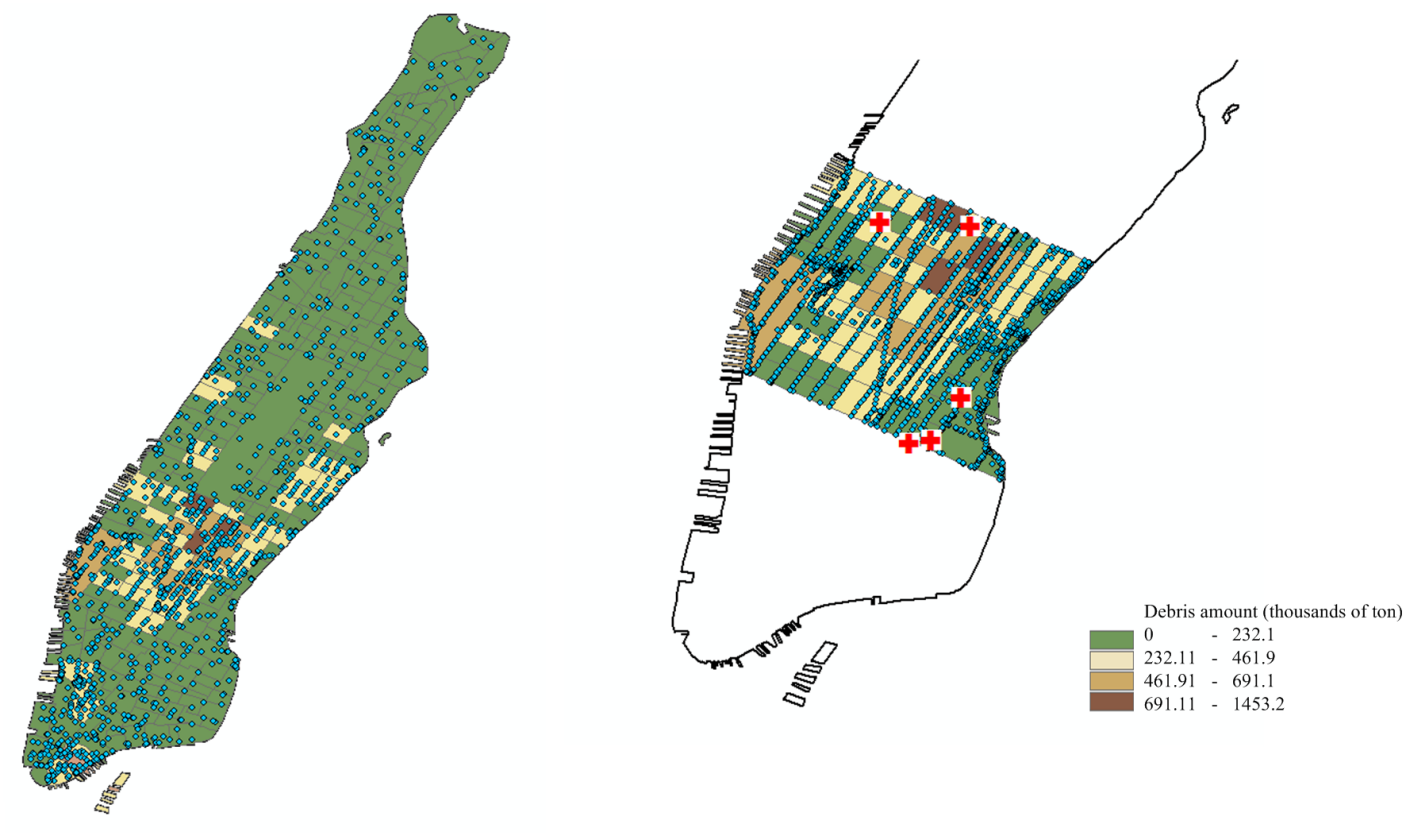
Manhattan after disruption. Debris amounts on the census tracts and resulting demand nodes from the sample earthquake in Fig 5 demonstrated on the middle and right panel (partial Manhattan instance), respectively. Maps are composed in ESRI ArcGIS 10.2.2.

Fig 5 demonstrates the road network of Boston and Manhattan along with hospital locations and a sample earthquake epicenter. Figs 6 and 7 display the estimated debris amounts on census tracts and the resulting demand nodes from the sample earthquake illustrated in Fig 5. We determine four levels of debris intensity on census tracts that are differentiated by color as indicated by the legend. In the given earthquake scenario, the highest amount of debris in Boston is accumulated in the downtown area, whereas in Manhattan the high debris tracts are distributed in the midtown and downtown areas.

In our experiments, we either use the entire Boston and Manhattan map (complete instances), or we extract a sub-section of the maps with similar structural characteristics for the two cities (partial instances). The right panels of Figs 6 and 7 illustrate the partial instances with debris distributions for Boston and Manhattan, respectively.

Table 1 summarizes the important topological and structural features and disruption based properties for Boston and Manhattan instances. The values pertaining to the disruption are the average for three different earthquake scenarios, indicated in Fig 4.

**Table 1:** Properties of Boston and Manhattan instances.

		Complete Instance		Partial Instance	
	Indicator	Boston	Manhattan	Boston	Manhattan
**Topological and Structural**	N	12,035	7,701	1,638	1,671
	E	16,854	12,131	2,280	2,617
	〈*k*〉	2.8	3.15	2.78	3.13
	|*N*_*S*_|	21	20	5	5
	*P*	617,059	1,572,562	72,662	287,283
**Disruption related**	Total debris	19,376	48,149	6,608	20,762
	$|\bar{E}|$ - (%)	8,488 - (50%)	9,327 - (76.8%)	2,280 - (100%)	2,617 - (100%)
	Total demand	13,929	42,117	12,735	23,941
	|*N*_*D*_| - (%)	794 - (6.6%)	709 - (9.2%)	1,638 -(≈ 100%)	1,671 - (≈ 100%)
	Total supply	14,217	42,971	12,992	24,422
	R	325	785	111	347
	*L*_*avg*_	50.64	195.88	47.64	168.47

First half of the table specifies the topological and structural properties of the networks that do not change with a disruption. The second half indicates the attributes that emerge as a result of the disruption. Notation: N = Number of nodes, E = Number of edges, 〈*k*〉 = Average degree, |*N*_*S*_| = Number of supply nodes, *P* = Population, $|\bar{E}|-\left(\%\right)$ = Number of blocked roads and its percentage over all the roads, |*N*_*D*_| − (%) = Number of demand nodes and its percentage over all the nodes, *R* = Amount of available clearance resources per period, *L*_*avg*_: Average shortest path distance between all node pairs: $\frac{1}{N(N-1)}\sum_{i\neq j}D_{ij}$, where *D*_*ij*_ is the shortest path distance between node *i* and *j* calculated with debris amounts as weights.

From Table 1, we observe that when the post-disruption effect is included in the calculation of demand nodes and blocked roads, as in the case of complete instances, the percentage of demand nodes and blocked roads is higher in Manhattan compared to Boston. This is mainly because of the geographical and structural properties (population, building types, occupancy, etc.) in Manhattan that lead to more debris and consequently to greater demand. However, for the partial instances, we distribute the debris on the roads and demand on the nodes in such a way that the percentage of demand nodes and debris-blocked roads are the same for Boston and Manhattan, which is almost ≈100%. This way, we exclude any difference in the instances that might result from debris or demand distribution.

## Results

### Validating the heuristic

In order to assess the quality of solutions generated by the Cent-Restore heuristic, we compare the solutions obtained via Cent-Restore to the RNRP-MIP solutions for the randomly generated instances. We solve RNRP-MIP with CPLEX 12.6.1 optimization solver. At each iteration CPLEX finds two values: (i) an upper bound (UB) on the optimal objective function value obtained by a relaxed solution, and (ii) a lower bound (LB) on the optimal objective function value obtained by the best feasible solution. The optimality gap of RNRP-MIP (*GAP*_*MIP*_) at any given time is computed as in Eq (14), and the gap decreases as these values converge to each other.

For a particular solution and its corresponding objective value *z*_*H*_ proposed by the Cent-Restore heuristic, the optimality gap for the heuristic (*GAP*_*H*_) can be calculated as in Eq (15).

$$\begin{array}{c} GAP_{MIP}=\frac{UB-LB}{LB}*100 \end{array}$$ (14)

$$\begin{array}{c} GAP_{H}=\frac{UB-z_{H}}{z_{H}}*100 \end{array}$$ (15)

Note that, if the optimization solver identifies (or verifies) an optimal solution, the optimality gap for RNRP-MIP becomes 0%. Then, the *UB* in *GAP*_*H*_ calculation becomes equal to the optimal objective function value. However, if the optimization solver is not able to identify an optimal solution in a given amount of time, *GAP*_*H*_ becomes an overestimate of the actual optimality gap since it is calculated with the upper bound on the optimal objective function value.

Table 2 summarizes the computational performance of Cent-Restore heuristic and RNRP-MIP in terms of the CPU time for instances with 80% blocked roads. Computational results for instances with 50% blocked roads are presented by S2 Table. We report two different time metrics for RNRP-MIP: time to prove optimality (Time (Opt.)) and time to find the best feasible solution (Time (B.)). In this way, we can observe whether the solver keeps improving the feasible solution over time or tries to prove an already found feasible solution as optimal by decreasing the upper bound. The maximum time we allow the solver to run is 24 hours.

**Table 2:** Computational performance for randomly generated networks when 80% of the roads are blocked with debris.

Grid Network	Supply Scenario	RNRP-MIP			Cent-Restore		Irregular Network	Supply Scenario	RNRP-MIP			Cent-Restore	
		Time (Opt.)	Time (B.)	GAP	Time	GAP			Time (Opt.)	Time (B.)	GAP	Time	GAP
**G(3, 20)**	**Distant**	24	6.12	19.2%	<1 s.	20.13%	**R(3, 20)**	**Distant**	8.5	0.1	0%	<1 s.	0.38%
	**Close**	24	0.15	21.26%	<1 s.	24.6%		**Close**	7.2	0.2	0%	<1 s.	0.8%
	**Multi-Finite**	24	0.11	13.25%	<1 s.	14.12%		**Multi-Finite**	10.2	0.3	0%	<1 s.	1.5%
	**Multi-Infinite**	24	0.8	7.34%	<1 s.	7.83%		**Multi-Infinite**	9.5	0.1	0%	<1 s.	0.9%
**G(5, 12)**	**Distant**	24	1.17	22.51%	<1 s.	25.6%	**R(5, 12)**	**Distant**	4.3	0.1	0%	<1 s.	0.9%
	**Close**	24	3.83	16.93%	<1 s.	17.5%		**Close**	4.6	0.08	0%	<1 s.	0.57%
	**Multi-Finite**	24	1.57	17.82%	<1 s.	19.11%		**Multi-Finite**	8.1	0.34	0%	<1 s.	0.71%
	**Multi-Infinite**	24	2.87	10.08%	<1 s.	11.52%		**Multi-Infinite**	5.6	0.12	0%	<1 s.	0.62%
**G(6, 10)**	**Distant**	24	6.15	17.91%	<1 s.	19.22%	**R(6, 10)**	**Distant**	3.4	<1 min.	0%	<1 s.	0.28%
	**Close**	24	3.64	21.15%	<1 s.	23.42%		**Close**	5.1	<1 min.	0%	<1 s.	0.4%
	**Multi-Finite**	24	3.26	11.83%	<1 s.	13.10%		**Multi-Finite**	6.7	<1 min.	0%	<1 s.	0.2%
	**Multi-Infinite**	24	1.09	9.10%	<1 s.	10.34%		**Multi-Infinite**	5.5	<1 min.	0%	<1 s.	0.1%

Grid network results are on the left side, irregular network results are on the right side. Unless otherwise indicated, the time units are in hours.

The experiments in this section are conducted on a computing cluster node with Intel E5 2650 CPU’s @ 2.00 GHz and 128 GB of RAM. We average the results over 10 replications for each instance type, which creates a total of 240 realizations.

As the results indicate, for grid networks the optimization solver fails to find a provably optimal solution within 24 hours. However, the time to find the best feasible solution is significantly less than 24 hours. Furthermore, the Cent-Restore is able to provide a *good* result based on the gap in less than 1 second. Optimality gaps for the RNRP-MIP and Cent-Restore heuristic are very close, suggesting that the heuristic solution is very close to the best feasible solution found by RNRP-MIP. Furthermore, the fact that the best found solution is not improved for a long time by RNRP-MIP may imply the optimal solution could be in the vicinity of the best feasible solution.

For irregular networks, the time for the solver to find a provably optimal solution is comparably less than grid networks, but larger than the time of Cent-Restore. We observe that the optimality gap for the heuristic solutions, calculated with the optimal objective function value, is less than 1%, except for one instance type.

In sum, we claim that for irregular networks, Cent-Restore provides near-optimal results within a second. For grid networks, within a second Cent-Restore finds solutions very close to the best feasible solution achieved by the RNRP-MIP in 24 hours. However, for these instance types optimality is not proven due to the underlying computational difficulty.

### Resilience implications

In order to evaluate the resilience of a CIS, we employ the framework proposed by Bruneau et al. [33]. We use the notion of *resilience curve*, a line plot, to demonstrate the evolution of a system’s functionality over time with respect to a quality metric. In this paper, we denote the metric by *Q*(*t*) and define it as the proportion of satisfied demand reached and served by supply nodes, varying between 0 and 1. This implies that the emergency service network returns to its fully functional state when *Q*(*t*) = 1. The time dimension for the *Q*(*t*) curve starts from a period where the system is fully functional, denoted by F, then the disruption occurs that causes *Q*(*t*) to drop down steeply. The period when the restoration operations begin is denoted by time 0. As Bruneau et al. suggests, the area under the resilience curves implies the degree of resilience. This way, resilience of the system is not characterized by a single value but rather by a process that involves multiple dimensions [33].

#### Randomly generated instances

**Fig 8:**
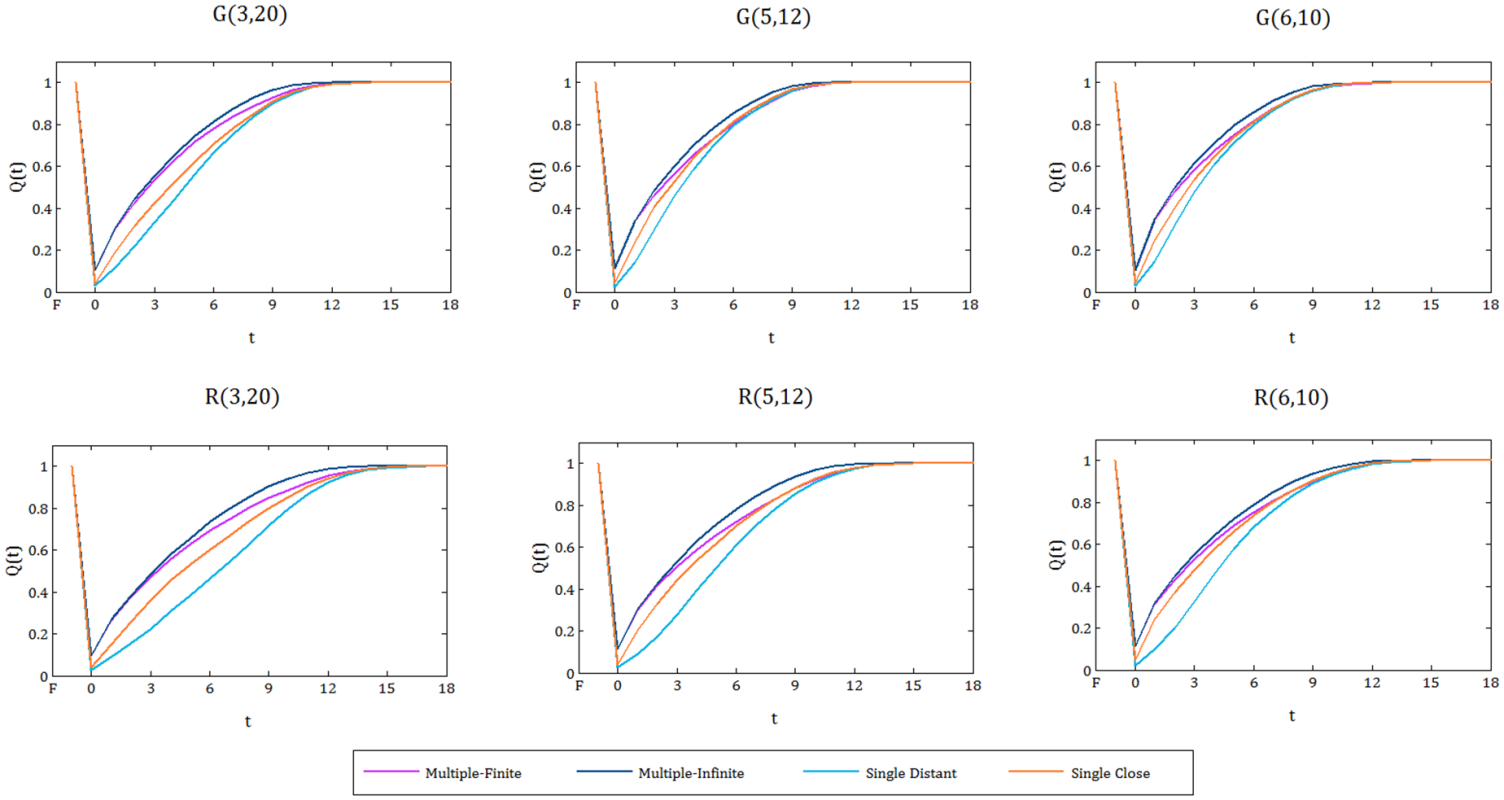
Resilience curves for grid and irregular networks with different structures. Top panels: Resilience curves for grid networks G(3, 20), G(5, 12), G(6, 10), from left to right. Bottom panels: Resilience curves for irregular networks R(3, 20), R(5, 12), R(6, 10), from left to right. In all of the graphs four different supply settings (single distant, single close, multiple-finite, and multiple-infinite) are represented.

For each randomly generated instance type (see Fig 1, e.g. Irregular, 3x20, Supply Distant, 80%) we generate 200 random disruption scenarios, and use Cent-Restore to establish a schedule for the restoration activities. The resulting percentage of satisfied demand over time is averaged over all scenarios and used as the quality metric in the resilience curves (Fig 8). Note that, we only include the instance types with 80% disruption, simply because for these instances the difference between them is clearer.

When we analyze the resilience curves in Fig 8 for each topology (grid or irregular), we observe that regardless of the network structure, having multiple supplies with a large capacity is the best supply setting in terms of resilience. Moreover, having a single supply point with a big or infinite capacity located in the most distant node of the network is the worst supply setting.

In all of the charts, the difference between single supply scenarios, distant and close, is remarkably large. This gap becomes even more evident in the irregular networks, and specifically for the 3x20 structured instances. Hence, as the shape of the network gets more elongated and the diameter of the network increases, the supply location has a bigger influence on resilience. To sum it all up, we claim that when the disruption is distributed uniformly at random, positioning of supply is a factor to improve resilience, and the extent of improvement depends on the structural features, especially the diameter, of the networks.

Furthermore, for the irregular networks, the quantity and location of supply makes a larger difference in the resilience of instances compared to the grid networks (the gaps between the resilience curves corresponding to different supply settings for irregular networks is more evident than for grid networks, see Fig 8). Hence, we can state that irregular networks are more sensitive to supply quantities and locations compared to grid networks.

**Fig 9:**
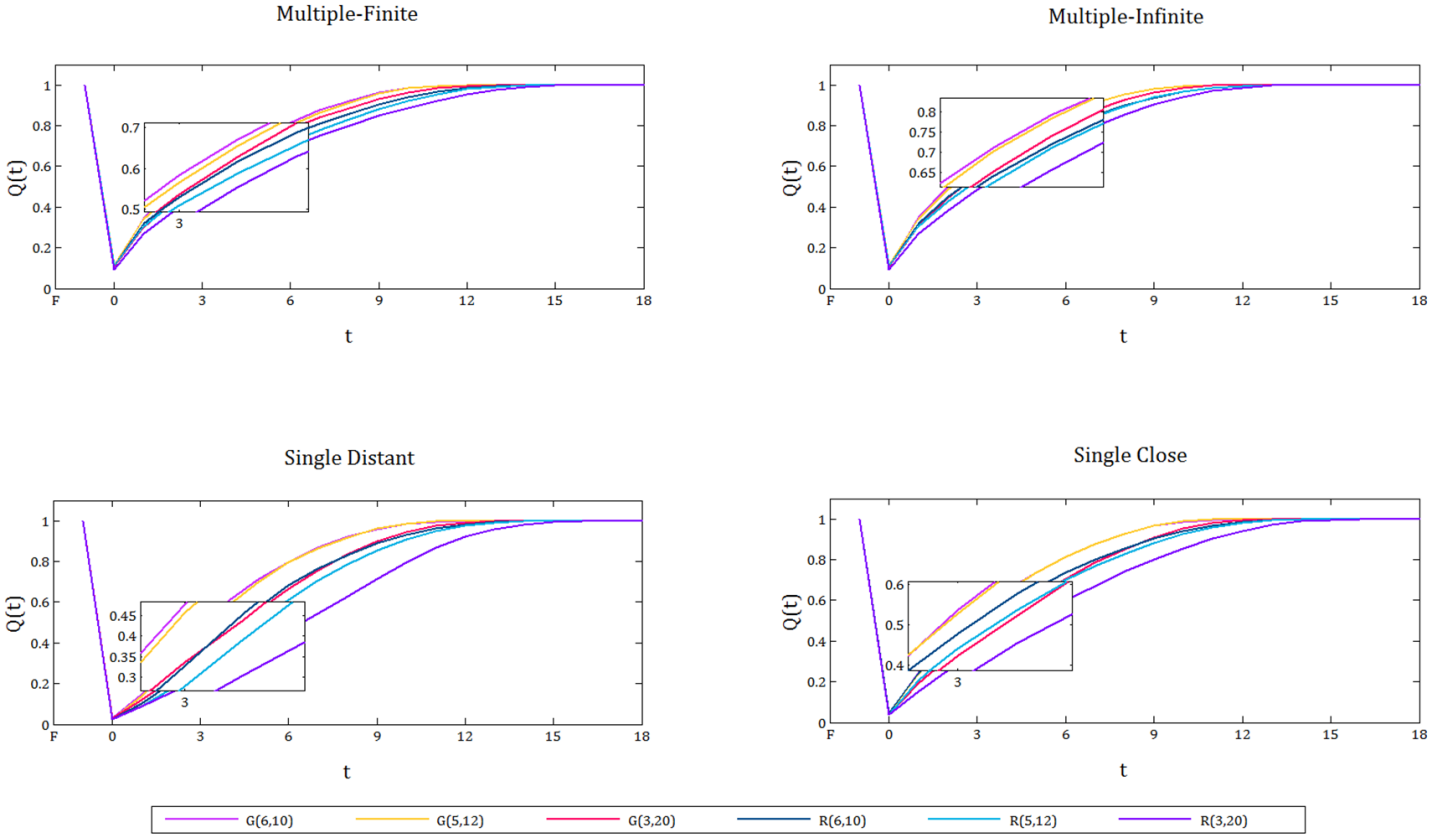
Resilience curves for each supply setting. Six different network instances are illustrated for each supply setting. The localized view suggests that grid networks are more resilient than the irregular networks that have the same structure.

Fig 9 demonstrates the resilience curves for each randomly generated instance under each supply setting. In a closer view, at each supply setting, for the networks with the same structure (e.g. G(3, 20) and R(3, 20)), we observe that, on average, the grid instance’s resilience curves are above the irregular instance’s resilience curves. Overall, we confirm that grid networks are more resilient than irregular networks. This claim is supported by a set of papers in the literature that study the network functionality after a random attack. They suggest that grid networks are more robust to disruptions than random irregular networks [30, 51].

#### Instances based on realistic settings

In this section, we illustrate the impact of various restoration strategies that are built on different prioritization rules the resilience of Boston and Manhattan. Furthermore, we explore the influence of the operational attributes of the overlaid emergency service network, the topology of the underlying road network, and the disruption distribution on the resilience.

**Fig 10:**
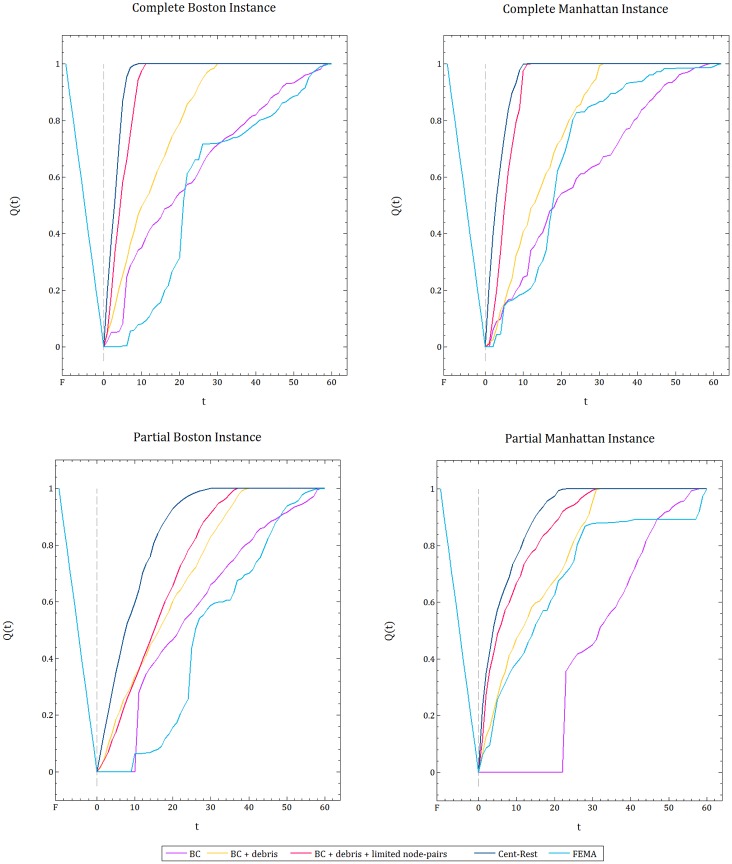
Resilience curves for different restoration strategies for the complete (on the upper panel) and partial (on the lower panel) Boston and Manhattan instances. Comparison of five different restoration strategies for the disruption scenarios on Manhattan (on the left side) and Boston (on the right side). Notation: BC = Road prioritization based on the generic betweenness centrality measure, BC + debris = BC *and* shortest paths in the BC measure are calculated with the debris amounts and the measure is scaled based on the shortest path value, BC + debris + limited node-pairs = BC + debris *and* BC measure is limited to supply and demand node pairs, Cent-Restore = Our heuristic for the RNRP problem; BC + debris + limited node-pairs *and* BC measure is scaled with supply and demand amounts. Time specified in the horizontal axis by *t* is in days.

Fig 10 illustrates the superiority of the Cent-Restore heuristic to four other restoration strategies for Boston and Manhattan instances. One of these strategies is based on FEMA’s recovery plan. In the most recent post-disaster guidelines FEMA provides [3], road clearance schedules are established by taking road attributes into account. Roads are prioritized for recovery based on their class: major arterial routes and primary highways are cleared first, local roads are cleared last. Based on our data, we group the roads into six main classes, with decreasing priority: primary highways, primary roads, secondary and connecting roads, local neighborhood and rural roads, roads with special characteristics, and other roads. We observe in Fig 10 that such prioritization of roads for recovery does not perform well with respect to resilience compared to the other restoration strategies.

The second strategy we implement is prioritization of restoration efforts based on the classical edge betweenness centrality; for an edge it is calculated as the summation of the generic node betweenness of its end nodes. Bhatia et al. claim that restoring disrupted components based on betweenness centrality connects the network faster than many other topology-based prioritization strategies, and has proven to be a very good strategy [34]. However, when the point of interest is the restoration of services and not merely re-establishing the connectivity within the network, prioritizing restoration efforts solely based on betweenness centrality (BC) emerges as one of the worst strategies, as depicted in Fig 10.

We accept BC as the simplest strategy, and create more complex strategies by improving the measure it uses, eventually leading to the Cent-Restore heuristic. First, we modify the measure used by BC by calculating the shortest paths using debris amounts rather than metric distances and scaling them by their shortest path value. We introduce a new restoration strategy based on this modified measure (BC + debris). As observed in Fig 10 this is a more effective strategy than BC, hence we claim that including the post-disruption attributes, such as disruption intensity (debris amounts) on the network components, on the recovery plan has a significant impact on resilience.

Next, on top of the first modification, we limit the node pairs accounted for while calculating the measure of BC to supply-demand node pairs. We display the resilience curves for this strategy (BC + debris +limited node-pairs) in Fig 10 and observe that creating a recovery plan based on the essential node pairs improves resilience even more.

Overall, we observe how the modifications on BC gradually improve the efficiency of the restoration strategies with respect to resilience, and the best strategy appears to be Cent-Restore, which includes weights for supply and demand amounts in the calculations. Hence, we conclude that including the operational characteristics of the service networks is crucial to improving resilience.

**Fig 11:**
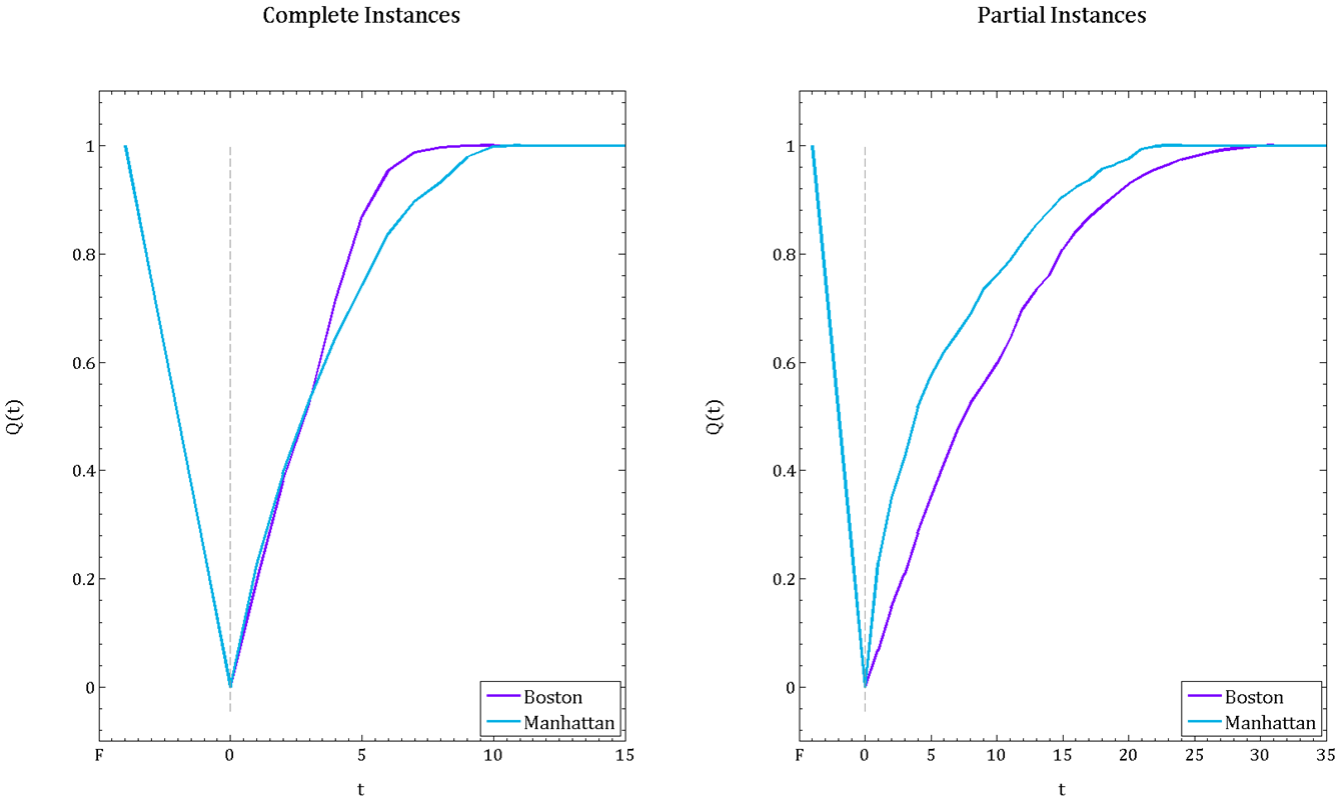
Resilience curves for Boston and Manhattan instances. Comparison of the resilience curves resulting from the Cent-Restore heuristic for the complete instance (left panel), and the partial instance (right panel).

Fig 11 illustrates the resilience curves for Cent-Restore for comparison of the Boston and Manhattan instances, when the complete and partial instances are used. Recall that, for the randomly generated instances when the disruption is distributed uniformly at random, we showed that the grid instances are more resilient than irregular instances. However, when we compare instances based on realistic settings (Fig 11, left panel), we observe that Boston recovers faster than Manhattan, which contradicts our previous claim. This is due to the fact that, in realistic settings, the distribution of the debris on the roads and the demand over the affected area are not distributed uniformly at random. In Table 1, we report the difference between the percentage of demand nodes and the percentage of blocked roads for the complete instances of Boston and Manhattan.

In the case of partial instances, we observe that Manhattan recovers faster than Boston (see Fig 11, right panel), which is aligned with our first statement. Recall that, when we extract a partition from the instances when there is a disruption, or when there is a demand, the distribution becomes uniform; 100% of the roads are blocked and 100% of the nodes are associated with a demand value. In addition, for the randomly generated instances that are included in the analysis, the percentage of demand is 50% and percentage of blocked roads is 80%. Taking all into account, we conclude that post-disruption characteristics (i.e. disruption distribution) are crucial to analyzing a network’s resilience. That is, in order to determine a city’s resilience we can not only work with pre-disruption attributes, but we need to perform a disruption scenario analysis and include the post-disaster impacts.

**Fig 12:**
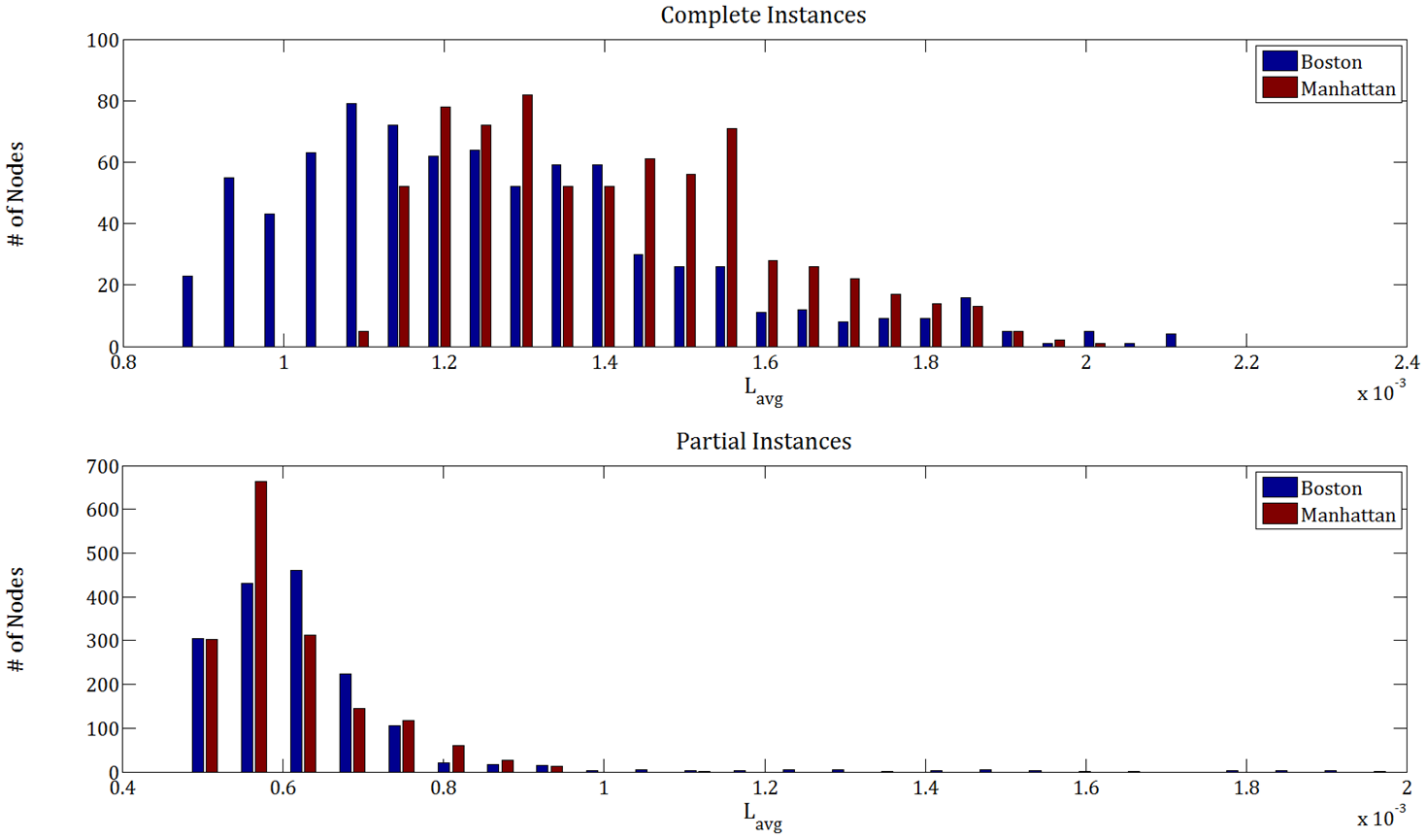
Distribution of the average distance between node-pairs. The average distance of demand node *i* to the rest of the nodes is calculated as: $L_{avg}^{i}=\frac{1}{\left(N-1\right)}\sum_{i\neq j}D_{ij}$ and normalized in order to compare different amounts in Boston and Manhattan data properly.

In order to explain the extent of the impact of the debris distribution on the resilience of different networks, we illustrate the rough distribution of average debris shortest path distance ($L_{avg}^{i}$) of a demand node *i* to all the other nodes in the network by plotting its histogram in Fig 12. This gives an effective understanding of the location of a demand node and whether it is easily reachable or not, due to its place inside a highly disrupted area. In a sense, it indicates how the debris is accumulated or dispersed. On the upper panel, when the disruption is on the complete instances, we observe that more nodes in the Boston network are easily reachable because they have a lower average distance. On the other hand, for the Manhattan network the distribution is peaked around the medium-distances, suggesting a less reachable demand node distribution than Boston. However, we observe that the histogram plots show difference for the partial instances. On the lower panel, we notice that there are more nodes in Manhattan that are easily reachable compared to Boston. These results elucidate the resilience curve performance (Fig 11) in which we observe a more resilient network for the partial Manhattan instance and complete Boston instance.

**Fig 13:**
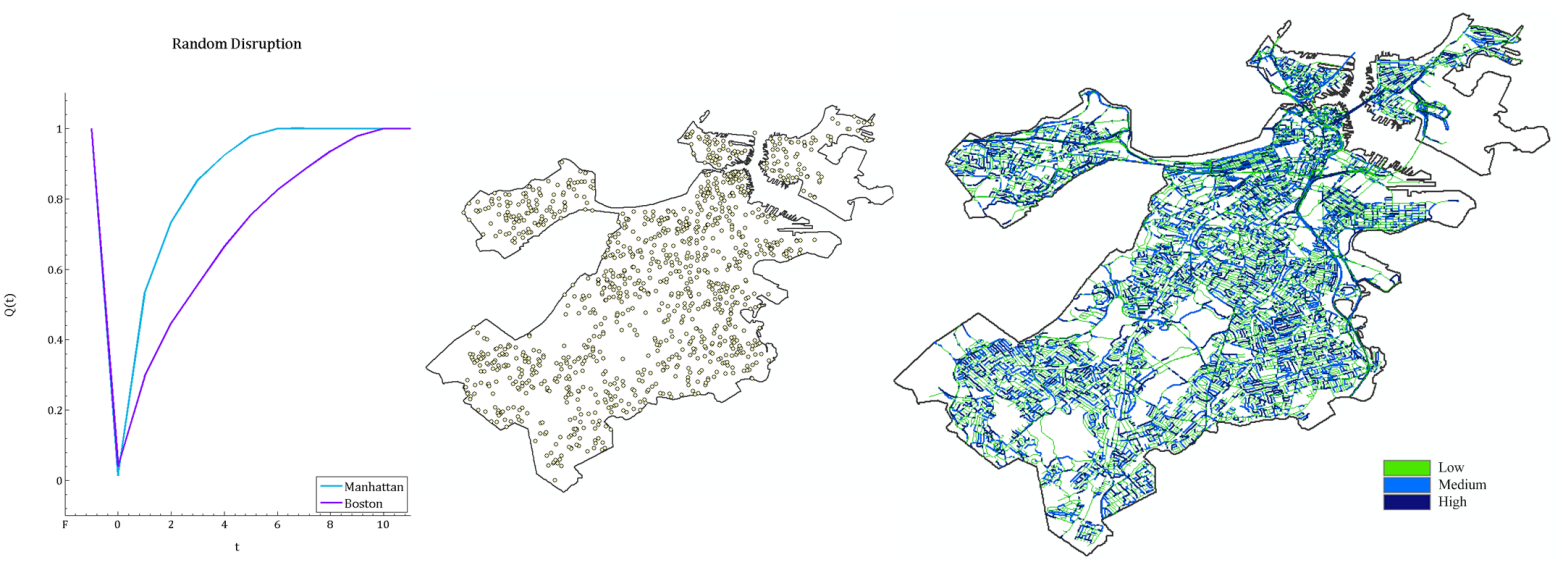
Resilience curves for the complete instances of Boston and Manhattan when the disruption is distributed uniformly at random, and an illustration of the post-disruption features on the Boston map. The resilience curves display the results averaged over 10 realizations (left panel). An example of a uniformly random disruption on the complete Boston map (a similar illustration can be imagined for Manhattan): (i) demand node locations and amounts are randomly assigned (middle panel); notice that they are not clustered with respect to the debris intensity as in Fig 6, (ii) 100% of the roads are blocked, debris amounts on them are generated randomly (right panel). As a road’s color gets darker and thicker, the debris on the roads increases.

To solidify this claim, we run additional experiments where the demand distribution and disruption is created uniformly at random on complete instances, and compare resulting resilience curves (averaging over 10 realizations) for the Cent-Restore. Fig 13 demonstrates that in such a disruption scheme, Manhattan (grid network) appears more resilient than Boston (irregular network).

**Fig 14:**
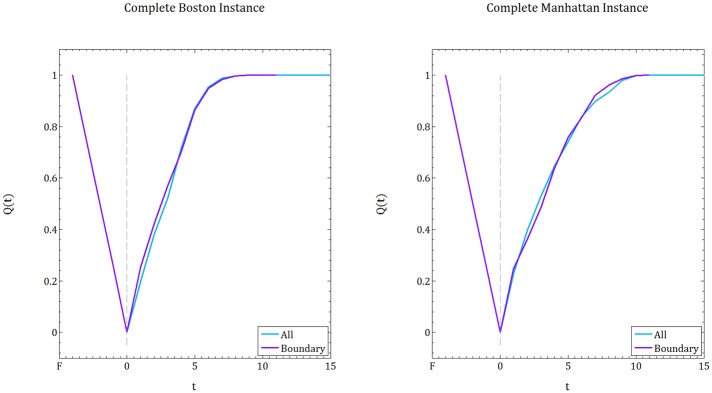
Impact of the supply locations to resilience. Comparison of the resilience curves when all of the supplies are intact and a subset of supply locations close to the boundary of the maps is selected for the Boston instance (left panel), and Manhattan instance (right panel). Resilience curves are an output of the Cent-Restore heuristic.

When discussing the randomly generated instances, we prove that the most distant nodes (metric distance) in the network are the worst locations for supply nodes. We experiment with a similar setting for Boston and Manhattan instances, and create a hypothetical scenario in which only the hospitals that are close to the boundaries of the maps are left as supply nodes (see Figs 7 and 6). The supply amount in these hospitals is scaled up to satisfy all the demand. Fig 14 displays the resilience curves resulting from Cent-Restore for the two supply scenarios: with all the hospitals and the other with a subset of hospitals that are close to the boundaries of the maps.

We observe that the difference between the curves is unnoticeable. This suggests that the metric distances of the road network are not enough to determine the goodness of supply locations, which was the case in randomly generated instances. Overall, we claim that the effectiveness of the supply position depends on the disruption, and the operational attributes of the disrupted service network.

To sum it all up, we show that analyzing post-disruption related characteristics of infrastructure systems are essential to develop an efficient recovery plan to restore services. Even though pre-planning is an important phase in disaster management, pre-known topological features of the networks are not sufficient to establish a good schedule for recovery efforts. Instead, learning the impact of the disruption and utilizing this knowledge in a fast heuristic to find a better schedule helps to restore functions of the networks faster. For the complete Boston and Manhattan instances, solving Cent-Restore takes an average of five and seven minutes, respectively. For the partial instances, this reduces down to one minute, on average, for both of the maps.

In addition, when the goal is to recommend restoration strategies for service networks, the results confirm that if the disruption is not distributed uniformly at random which is the case in realistic settings, the topological properties of the underlying infrastructure networks lose their significance, and the operational attributes of the overlaying disrupted service network gain importance. Hence, it is not possible to label a network as more resilient by merely looking at its topological structure, which is the case in most of the existing literature.

## Discussion

In this paper, we integrated ideas from the field of network science and operations research to establish effective recovery plans to improve the resilience of critical infrastructure systems. In addition, we provide insights on how the information on the impact of a disruption and the operational and topological attributes of service networks benefit the resilience analysis.

We propose a novel centrality measure, SNEBC, to assess the criticality of the disrupted service network components, and use this measure to develop a heuristic, Cent-Restore, for building a restoration schedule for disrupted CIS.

As an illustrative case study, we explore the road network recovery problem (RNRP) in the context of post-disaster response and recovery. In this case, the disruption is caused by debris on the roads that obstructs accessibility in the network. The goal is to re-establish the connectivity of medical care facilities and disaster survivors to enable the flow of services in a timely manner by clearing the roads.

For this problem, we use two types of instances: (i) randomly generated instances, and (ii) instances based on realistic settings, Boston and Manhattan. For randomly generated instances, we prove that Cent-Restore provides near-optimal results in a comparably short time. For the realistic instances, we evaluate the performance of Cent-Restore with respect to resilience by comparing it with other restoration strategies that are based on different prioritization rules of the disrupted components. We show that Cent-Restore re-establishes the services faster than other considered strategies. This is because, Cent-Restore incorporates the service and post-disruption related characteristics of the networks while generating a restoration schedule.

In addition, we demonstrate that if the disruption is distributed uniformly at random, grid networks are more resilient than irregular networks. Positioning supply locations far away (in terms of metric distance) from the rest of the nodes appears to greatly hamper resilience. In addition, supply capacities and positions are more important for irregular networks.

However, in realistic settings, disruption intensity varies over the network, hence the distribution of the disruption and demand locations vary. When this is the case, we conclude that using the topological attributes of the underlying infrastructure networks is not sufficient to derive effective restoration strategies. The distribution of the disruption on the overlaying service network and the location and capacity of the service components (such as demand and supply) within the disrupted network is more important. As we observe in Fig 10, considering the post-disruption and service related components in the restoration strategies gradually improves resilience. Also, we observe how topological attributes of the networks lose their significance when it comes to determining good locations for the supply points (Fig 14).

In sum, either the post-disruption effects could be forecasted in the pre-planning phase and an optimal restoration schedule can be sought, which is computationally cumbersome, or the real impact of the disruption could be learned in the response phase and utilized in a fast heuristics (such as Cent-Restore) to generate a near-optimal restoration schedule.

As a potential future work, we consider disruption scenarios on interdependent networks. Study of interdependent infrastructures is crucial since many CIS are highly interconnected and a failure on any of these systems can lead to cascading failures and may have severe results. The goal is to extend the centrality measure we present in this study by incorporating the characteristics of these interdependent networks and provide an efficient restoration strategy for the entire interconnected system.

## Supporting information

S1 Fig The benefit function, *b*^*t*^. The function is calculated with parameters: λ = 0.2 and *C* = 120. The value of the function for each time period *t* ∈ *T* is the benefit parameter *b*^*t*^ in the objective function of RNRP-MIP. For illustration, the planning horizon is limited to the first 20 periods.
(TIF)

S1 Appendix Proof of Theorem 1. (PDF)

S2 Appendix Instructions on how to create the randomly generated instances. (PDF)

S1 Table Notation for RNRP-MIP. Any time period referred as t belongs to the set *T*; *t* ∈ *T*.
(PDF)

S2 Table Computational performance for randomly generated networks when 50% of the roads are blocked with debris. Grid network results are on the left side, irregular network results are on the right side. Unless otherwise indicated, the time units are in minutes.
(PDF)
