# Supplementary figures and images for: Restoration of services in disrupted infrastructure systems: A network science approach
Source: PLoS One. 2018 Feb 14;13(2):e0192272. doi: 10.1371/journal.pone.0192272 (PMC5812613; doi:10.1371/journal.pone.0192272)

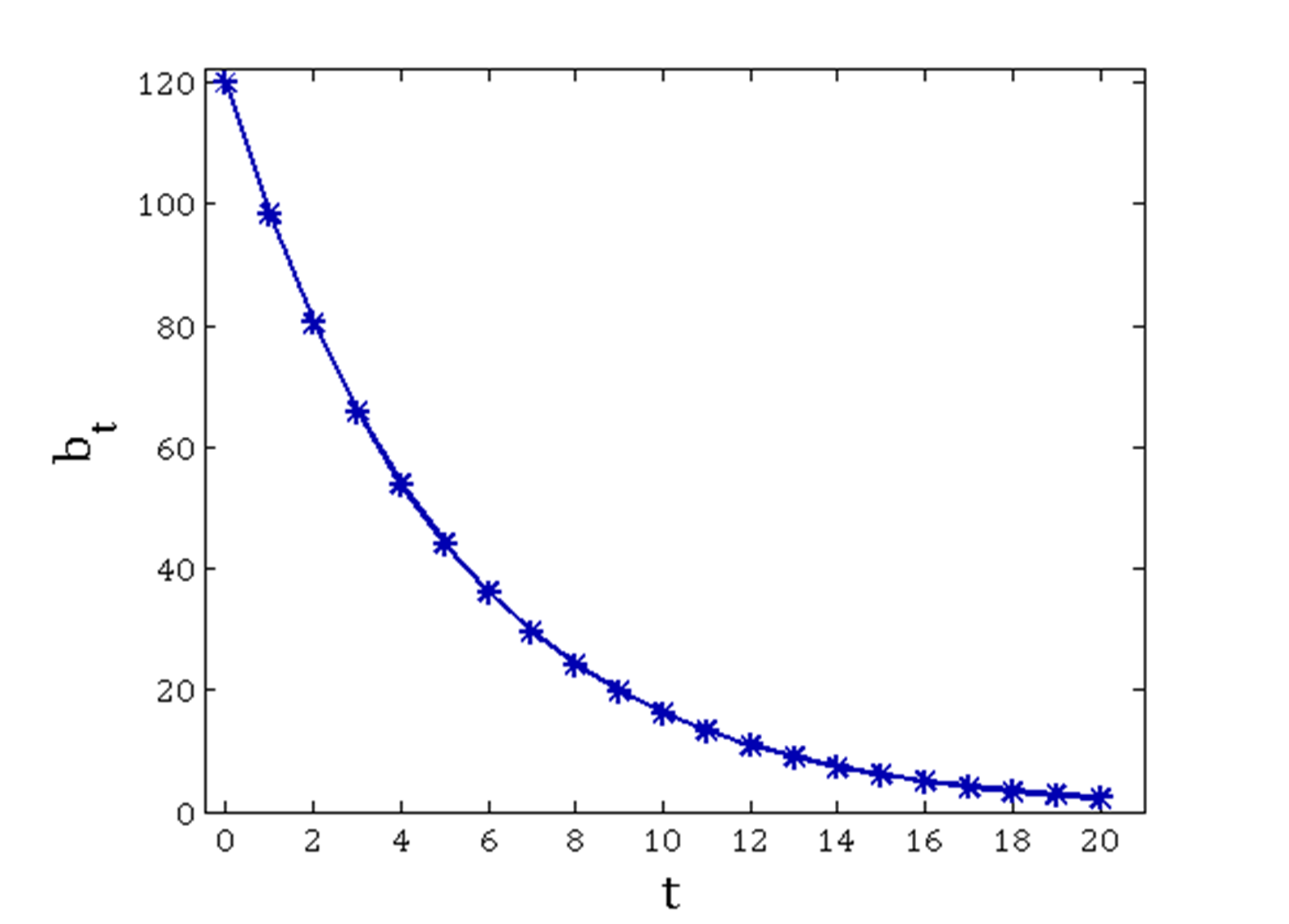

Supplement: S1 Fig — The function is calculated with parameters: λ = 0.2 and C = 120. The value of the function for each time period t ∈ T is the benefit parameter bt in the objective function of RNRP-MIP. For illustration, the planning horizon is limited to the first 20 periods. (TIF) [file pone.0192272.s001.tif]
