# Supplementary material for: Restoration of services in disrupted infrastructure systems: A network science approach
Source: PLoS One. 2018 Feb 14;13(2):e0192272. doi: 10.1371/journal.pone.0192272 (PMC5812613; doi:10.1371/journal.pone.0192272)
Supplement: S1 Appendix — (PDF) [file pone.0192272.s002.pdf]

Proof. Take an optimal solution $\mathcal{S}^*$ generated by RNRP-MIP, represented as the collection of the set of roads to be cleared ($\mathcal{S}_t$) and the amount of clearance completed on them for each discrete time period $t \in T$. Note that, the solution does not explicitly state the clearing order of the roads in $\mathcal{S}_t$ and the set $\mathcal{S}_t$ may consist of roads that become reachable due to the previously cleared roads within the same time period t. Therefore when we refer to a road being unreachable at time t we assume that even after all the scheduled clearance during time t (in addition to the clearance completed in the periods before t) that road still is not connected to the clearance resources through an unblocked path.

For all $t \in T$, if all the roads that start to get cleared at time t are reachable from clearance resources located at supply nodes, then the optimal solution itself corresponds to the equivalent solution in the statement, which completes the proof.

Otherwise, let us assume that there exists a blocked road $\{i,j\}$ that starts to get cleared at time t and unreachable from clearance resources located at supply nodes at time t, with a resource requirement of $W_{i,j}$. There are two possible cases: either $\{i,j\}$ becomes reachable at some $\bar{t} : \bar{t} > t$, or it never becomes reachable. If it never becomes reachable, $\{i,j\}$ is deleted from all the sets of roads it appears. Deleting road $\{i,j\}$ does not decrease the objective function hence we obtain a solution equivalent to the one in the statement. Otherwise pick the path from supply node s to $\{i,j\}$ that gets completely cleared in $\mathcal{S}^*$ at time $\bar{t} : \bar{t} > t$, such that the clearance time $\bar{t}$ for this path is the earliest among all such paths. Let $\mathcal{E} = \{e_1, e_2, e_3, ...\}$ be the set of roads on this path that get completely cleared after time t and let the roads in $\mathcal{E}$ are indexed in the order of closeness to the supply node s. Pick the closest road to s, $e_1 \in \mathcal{E}$ with a resource requirement of W_{e_1} that gets completely cleared at time $t' : t' > t$.

Now, let us create a new solution $\bar{\mathcal{S}}$ by switching the clearance order of the time periods where e_1 and $\{i,j\}$ are cleared such that the time that e_1 is completely cleared is before or at the same time period as $\{i,j\}$ starts to get cleared. An important consideration is to keep the same magnitude of clearance activity at each time period as before so that the clearance time of the other roads will not be affected. Fig 1 illustrates an example of how the clearance order of e_1 and $\{i,j\}$ are switched so that the clearance start time of $\{i,j\}$ is after or at the same time e_1 is completely cleared. The same argument is applied recursively by selecting the next road from the set $\mathcal{E}$ until $\{i,j\}$ starts to get cleared at or after $\bar{t}$. Such a switch can not improve the objective function since that would contradict the fact that $\mathcal{S}^*$ is an optimal solution. Also it will not worsen the objective function for the following reasons: (i) the clearance completion time for all the roads that are not in $\mathcal{E}$ do not change, (ii) the clearance completion time for all the roads that are in $\mathcal{E}$ either stays the same or becomes earlier, and (iii) the clearance completion time of road $\{i,j\}$ may be later, however the time that it becomes reachable stays the same (t') in both $\mathcal{S}^*$ and $\bar{\mathcal{S}}$, and a road can not contribute to the objective function by sending flow if it is not reachable. Hence $\bar{\mathcal{S}}$ corresponds to the equivalent solution in the statement. □

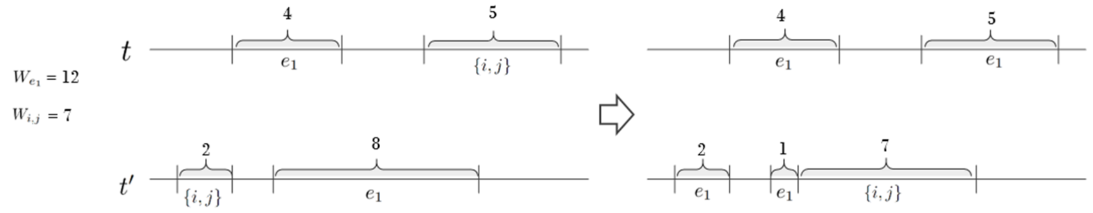

Figure 1: **An example for switching the times of clearance activities between $\{i,j\}$ and e_1 where $t' > t$.** Left side of the arrow is the possible clearance activities in the optimal solution $\mathcal{S}^*$ at time t and t' and right side is the rearranged clearance activities in the solution $\bar{\mathcal{S}}$ at time t and t'.
