# Supplementary material for: Restoration of services in disrupted infrastructure systems: A network science approach
Source: PLoS One. 2018 Feb 14;13(2):e0192272. doi: 10.1371/journal.pone.0192272 (PMC5812613; doi:10.1371/journal.pone.0192272)
Supplement: S2 Appendix — (PDF) [file pone.0192272.s003.pdf]

Instructions for Creating the Randomly Generated Instances

Randomly generated instances are divided into two groups based on their topology: grid and irregular. Each network is further divided into three subgroups based on their structures that determine their shape: 3x20, 5x12, and 6x10. All of the networks are set to have 60 nodes and half of the nodes (30) are specified as demand points with an unit demand. We would like to remark to the reader that some of these demand nodes may be connected to a supply node through an unblocked path at the beginning of the planning horizon. In this case, we assume that those demand points are served immediately at the beginning of the planning horizon as the result of a preprocessing step and they are not considered as demand nodes in the model. In other words, the total demand varies based on the disruption scenario. Additionally, we defined two disruption scenarios; either 50% or 80% of the roads are blocked with debris.

For all random number generation operations we used Mersenne Twister generator and we provide the corresponding seed numbers for the different replications below (inside parenthesis.)

Grid:

Creating a grid network with a particular structure is trivial, 60 nodes are positioned equidistantly within the specified area. For instance, for 6x10 structure we can imagine a grid network with 6 row nodes and 10 column nodes (see Fig 2). Hence, for each network type the number of edges is fixed. There are 97, 103, and 104 edges for 3x20, 5x12, and 6x10 structured networks, respectively.

For each network type (e.g. Grid 3x20), we defined four scenarios varying on location and amount of the supplies. First supply scenario,*single distant,* sets one of the four corner nodes of the grid network to be a supply point with a big or infinite capacity. Second scenario, *single close*, sets one of the middle nodes of the grid network to be a supply point with a big or infinite capacity. Third and fourth scenarios are named *multiple finite* and *multiple infinite*, respectively. For both of these scenarios we used the same three randomly located supply nodes but vary the amount of supply at the nodes and analyze the effect of the supply capacity. Specifically, for all instances of 3x20 the three supply nodes are located at the following row and column pairs: $(1, 17), (2, 2)$, and $(3, 10)$. Similarly, for 5x12, supply nodes are located at: $(1, 7), (3, 2)$, and $(5, 4)$. Finally, for 6x10, supply nodes are located at: $(1, 3), (1, 9)$, and $(5, 5)$. For multiple finite supply scenarios, the total supply capacity is defined to be just enough to cover all the demand and divided equally among the supply nodes. For instance, if the total demand is 28 all the three supply nodes have a supply capacity of 10. For the multiple infinite supply scenario all the supply nodes are set to have a big or infinite capacity.

For each network type and supply scenario (e.g. Grid 3x20, Single Close), we created 10 random instances by varying the demand nodes within the network (seed numbers: 5,10,15,20,25,30,35,40,45,50). Finally we defined 100 replications for each network type (seed numbers: 1 to 100), supply and demand scenario combination. These replications vary based on the distribution of the disruption; for each disruption percentage (50% and 80%) blocked roads are chosen randomly and the amount of debris on them varies uniformly at random from 1 to the per period clearance resources available. We ensured that ratio of the total amount of debris to the available clearance resources per period is the same for each network type. In total, we created a total of $24,000$ realizations.

Irregular:

For any irregular network with a particular structure, we used a Poisson point process to randomly scatter the 60 nodes within the rectangular area defined by its structure. We set the number of edges equal to the number of edges of grid networks with the same structure. For example, irregular and grid networks with a structure of 3x20 have a total of 97 edges. Then we randomly placed the edges in between nodes by taking into account the planarity requirements of the network. We

defined half of the nodes to be demand points. We replicated this process 10 times (seed numbers: 4,8,14,16,36,77,82,88,99,101) for each network type, which created a total of 30 instances in total. Similar to the grid networks, for each of these instances we used the same four supply scenarios. For each instance, the most and least distant nodes are calculated for the *single distant* and *single close* supply scenario, respectively. For the multiple supply scenarios, three supply nodes are selected randomly based on the provided seed numbers. Then we further created 100 replications (seed numbers: 1 to 100) for each disruption percentage. This way we created a total of $24,000$ realizations.
