# Supplementary material for: Restoration of services in disrupted infrastructure systems: A network science approach
Source: PLoS One. 2018 Feb 14;13(2):e0192272. doi: 10.1371/journal.pone.0192272 (PMC5812613; doi:10.1371/journal.pone.0192272)
Supplement: S1 Table — Any time period referred as t belongs to the set T; t ∈ T. (PDF) [file pone.0192272.s004.pdf]

Parameters	
b^t	Benefit accrued at period t by satisfying a unit of demand
R_t	Aggregate amount of clearance resources at period t
$W_{i,j}$	Amount of clearance resources required to fully clear edge $\{i,j\} \in \bar{E}$
$\mathcal{M}$	A big number
Decision Variables	
ud_j^t	Remaining unsatisfied demand at node $j \in N_D$ at the *end* of period t
d_j^t	Satisfied demand at node $j \in N_D$ *during* period t
rs_i^t	Remaining supply at node $i \in N_S$ at the *end* of period t
s_i^t	Amount of allocated supply from node $i \in N_S$ *during* period t
$f_{i,j}^t$	Amount of flow on edge $\{i,j\} \in E$ *during* time t
$A_{i,j}^t$	Amount of clearance completed on edge $\{i,j\} \in \bar{E}$ *until* the *end* of period t
$y_{i,j}^t$	Amount of clearance done on edge $\{i,j\} \in \bar{E}$ *during* period t
$\gamma_{i,j}^t$	Binary variable indicating if edge $\{i,j\} \in \bar{E}$ is fully cleared or not at the *end* of t
