# Supplementary material for: Restoration of services in disrupted infrastructure systems: A network science approach
Source: PLoS One. 2018 Feb 14;13(2):e0192272. doi: 10.1371/journal.pone.0192272 (PMC5812613; doi:10.1371/journal.pone.0192272)
Supplement: S2 Table — Grid network results are on the left side, irregular network results are on the right side. Unless otherwise indicated, the time units are in minutes. (PDF) [file pone.0192272.s005.pdf]

Grid Network	Supply Scenario	RNRP-MIP Time (Opt.)	RNRP-MIP Time (B.)	RNRP-MIP GAP	Cent-Restore Time	Cent-Restore GAP	Irregular Network	Supply Scenario	RNRP-MIP Time (Opt.)	RNRP-MIP Time (B.)	RNRP-MIP GAP	Cent-Restore Time	Cent-Restore GAP
**G(3,20)**	**Distant**	10.21	0.54	0 %	< 1 s.	0.011 %	**R(3,20)**	**Distant**	0.51	0.058	0 %	< 1 s.	0.3 %
	**Close**	10.87	0.32	0 %	< 1 s.	0.015 %		**Close**	0.42	0.02	0 %	< 1 s.	0.45%
	**Multi-Finite**	5.1	0.12	0 %	< 1 s.	0.2 %		**Multi-Finite**	0.82	0.12	0 %	< 1 s.	0.75 %
	**Multi-Infinite**	3.66	1	0 %	< 1 s.	0.1 %		**Multi-Infinite**	0.42	0.15	0 %	< 1 s.	0.091%
**G(5,12)**	**Distant**	7.4	0.27	0 %	< 1 s.	0.01 %	**R(5,12)**	**Distant**	0.25	0.167	0 %	< 1 s.	0%
	**Close**	5.5	0.23	0 %	< 1 s.	0.01%		**Close**	0.25	0.167	0 %	< 1 s.	0 %
	**Multi-Finite**	6.8	1.10	0 %	< 1 s.	0.14%		**Multi-Finite**	0.22	0.143	0 %	< 1 s.	0.07 %
	**Multi-Infinite**	4.2	0.77	0 %	< 1 s.	0 %		**Multi-Infinite**	0.22	0.15	0 %	< 1 s.	0.042 %
**G(6,10)**	**Distant**	1.5	0.015	0 %	< 1 s.	0.04 %	**R(6,10)**	**Distant**	0.15	0.042	0 %	< 1 s.	0%
	**Close**	1.8	0.045	0 %	< 1 s.	0.2 %		**Close**	0.051	0.016	0 %	< 1 s.	0.002 %
	**Multi-Finite**	0.95	0.022	0 %	< 1 s.	0.51 %		**Multi-Finite**	0.35	0.047	0 %	< 1 s.	0.001 %
	**Multi-Infinite**	1.2	0.088	0%	< 1 s.	0.31 %		**Multi-Infinite**	0.24	0.016	0 %	< 1 s.	0%
